# New C8-substituted caffeine derivatives as promising antioxidants and cytoprotective agents in human erythrocytes

**DOI:** 10.1038/s41598-022-27205-8

**Published:** 2023-01-31

**Authors:** Arleta Sierakowska, Beata Jasiewicz, Łukasz Piosik, Lucyna Mrówczyńska

**Affiliations:** 1 Department of Bioactive Products, Faculty of Chemistry, Adam Mickiewicz University in Poznań, Uniwersytetu Poznańskiego 8, 61-614 Poznań, Poland; 2 Department of Cell Biology, Faculty of Biology, Adam Mickiewicz University in Poznań, Uniwersytetu Poznańskiego 6, 61-614 Poznań, Poland

**Keywords:** Biochemistry, Biological techniques, Biotechnology, Cell biology, Chemical biology, Computational biology and bioinformatics, Drug discovery, Molecular biology, Health care, Health occupations, Risk factors, Chemistry

## Abstract

New structurally diverse groups of C8-substituted caffeine derivatives were synthesized and evaluated for their chemical and biological properties. Mass spectrometry, FT-IR, and NMR characterizations of these derivatives were performed. The cytotoxic activity of the derivatives was estimated in vitro using human red blood cells (RBC) and in silico pharmacokinetic studies. The antioxidant capacity of the compounds was analyzed using a ferrous ion chelating activity assay. The ability of the derivatives to protect RBC from oxidative damage, including the oxidation of hemoglobin to methemoglobin, was assessed using a water-soluble 2,2′-azobis(2-methyl-propionamidine) dihydrochloride (AAPH) as a standard inducer of peroxyl radicals. The level of intracellular oxidative stress was assessed using the fluorescent redox probe 2′,7′-dichlorodihydrofluorescein diacetate (DCF-DA). The results indicate that all derivatives are biocompatible compounds with significant antioxidant and cytoprotective potential dependent on their chemical structure. In order to explain the antioxidant and cytoprotective activity of the derivatives, a mechanism of hydrogen atom transfer (HAT), radical adduct formation (RAF), or single electron transfer (SET), as well as the specific interactions of the derivatives with the lipid bilayer of RBC membrane, have been proposed. The results show that selected modifications of the caffeine molecule enhance its antioxidant properties, which expands our knowledge of the structure–activity relationship of caffeine-based cytoprotective compounds.

## Introduction

Reactive oxygen species (ROS) are constitutively generated during metabolic processes in every cell and play an important role in signal transduction. An imbalance between ROS generation and cellular antioxidant defense leads to oxidative stress, contributing to the development of civilization diseases, including cancer and cardiovascular diseases^1^. Therefore, the balance between the formation of ROS and their elimination by scavenging systems plays a key role in cell physiology. In this approach, both natural and synthetic antioxidants have received much attention from a pharmaceutical and food chemistry viewpoint due to their proven health-promoting effects^2–5^.

Caffeine (1,3,7-trimethylxanthine) is one of the most important purine alkaloids with interesting pharmacological properties, including antioxidant capacity^6–8^. León-Carmona and Galano proposed five mechanisms of caffeine reaction with ROS, namely, radical adduct formation (RAF), hydrogen atom transfer (HAT), single electron transfer (SET), sequential electron proton transfer (SEPT), and proton coupled electron transfer (PCET)^9^. Finally, RAF has been identified as the main mechanism involved in the direct scavenging effect of caffeine; however, the type of ROS, as well as the polarity of the environment, may modify the mechanism. It should be mentioned that caffeine is a good scavenger of the highly reactive hydroxyl radical (^·^OH)^10^.

The hydroxyl radical is the most oxidizing agent that attacks most organic molecules and is intensively studied due to its importance in biological and environmental processes^11^. The ^·^OH radical is formed by the Fenton reaction between ferrous ions and hydrogen peroxide (Fe^2+^  + H_2_O_2_ → Fe^3+^  + ^·^OH + OH^−^), so the ratio of hydrogen peroxide to Fe^2+^ affects the generation of ^·^OH. On the other hand, iron is very important in many metabolic processes and is essential for the synthesis of hemoglobin (Hb) during erythropoiesis. Hb is the main protein component in red blood cells (RBC) and is essential for oxygen binding and transfer, while impaired iron metabolism causes various diseases, including cancer^12,13^. Therefore, iron chelation has already been proposed as a new strategy for cancer treatment, and several iron chelators have been developed for this purpose^14–17^. Caffeine is a weak iron chelator^18^, but we have previously described novel di- and polyamine caffeine analogs with significantly higher ferrous ions chelating activity than caffeine^19^.

RBC are the main cellular constituent of human blood and, during their life span (~ 120 days) in the circulatory system, are particularly exposed to both endogenous and exogenous compounds, including ROS^20^. Due to the presence of Hb and high levels of polyunsaturated lipids in the cell membrane, the harmful effects of ROS are most pronounced in RBC compared to other cell types. Therefore, the effect of oxidative stress on the shortening RBC lifespan by ROS-dependent eryptosis has been described and antioxidants have been identified as antieryptotic and and antianemic agents against many systemic diseases^21^. Enzymatic antioxidants such as catalase, superoxide dismutase, and glutathione peroxidise minimize the damaging effects of ROS, but the ability of RBC to neutralize ROS is limited. Therefore, molecules with antioxidant properties are used by RBC to improve their survival under oxidative stress^22,23^. It has been confirmed that dietary antioxidants, which have the ability to incorporate into the cell membrane, can protect Hb from oxidation to methemoglobin (MetHb)^24^. This is very important from physiological point of view, because MetHb with oxidized ferrous iron (Fe^2+^) to ferric iron (Fe^3+^), is unable to bind and transport oxygen (O_2_) to tissues and consequently acute or chronic hypoxia can occur. Hypoxia plays a role in the pathogenesis of major causes of mortality, including cancer, metabolic diseases, chronic heart and kidney diseases and myocardial ischemia^25^. The effects of long-term hypoxia on RBC membrane properties and blood viscosity have been also described^26^. In addition, significantly elevated MetHb levels in blood of severely ill COVID-19 patients, have recently been reported^27^. Caffeine has also been suggested to have health benefits in relation to SARS-CoV-2 infection, both directly and indirectly, by promoting immunomodulation, bronchodilation and inhibition of intracellular viral transcription^28^. Moreover, caffeine crosses the RBC cell membrane and scavenges the most harmful hydroxyl radical, preventing caspase-3 activation and MetHb formation^29^. The formation of caffeine-Hb complexes, stabilized by hydrophobic and hydrogen bond interactions, has been confirmed by in vitro^30^ and in silico^29^ studies. Interestingly, the caffeine metabolite 1-methyluric acid inhibits nitrite-induced Hb oxidation better than caffeine^31^. In conclusion, caffeine, its metabolites and derivatives are therefore interesting antioxidants with protective effects against Hb oxidation, which may find various biomedical applications. Furthermore, RBC lacking nuclei and other organelles provide a convenient model for in vitro evaluation of the interaction of natural and synthetic compounds with the cell membrane and Hb, respectively^32–34^.

The aim of this study was to synthesize new C8-substituted caffeine derivatives and evaluate their efficiency as iron chelators and cytoprotective agents under oxidative stress induced by peroxyl radicals generated from 2,2′-azobis (2-methyl-propionamidine) dihydrochloride (AAPH) in human RBC. A mechanism for the cytoprotective effects of the new derivatives in RBC under oxidative stress has been proposed.

## Results and discussion

### Synthesis of new caffeine derivatives

**Figure 1:**
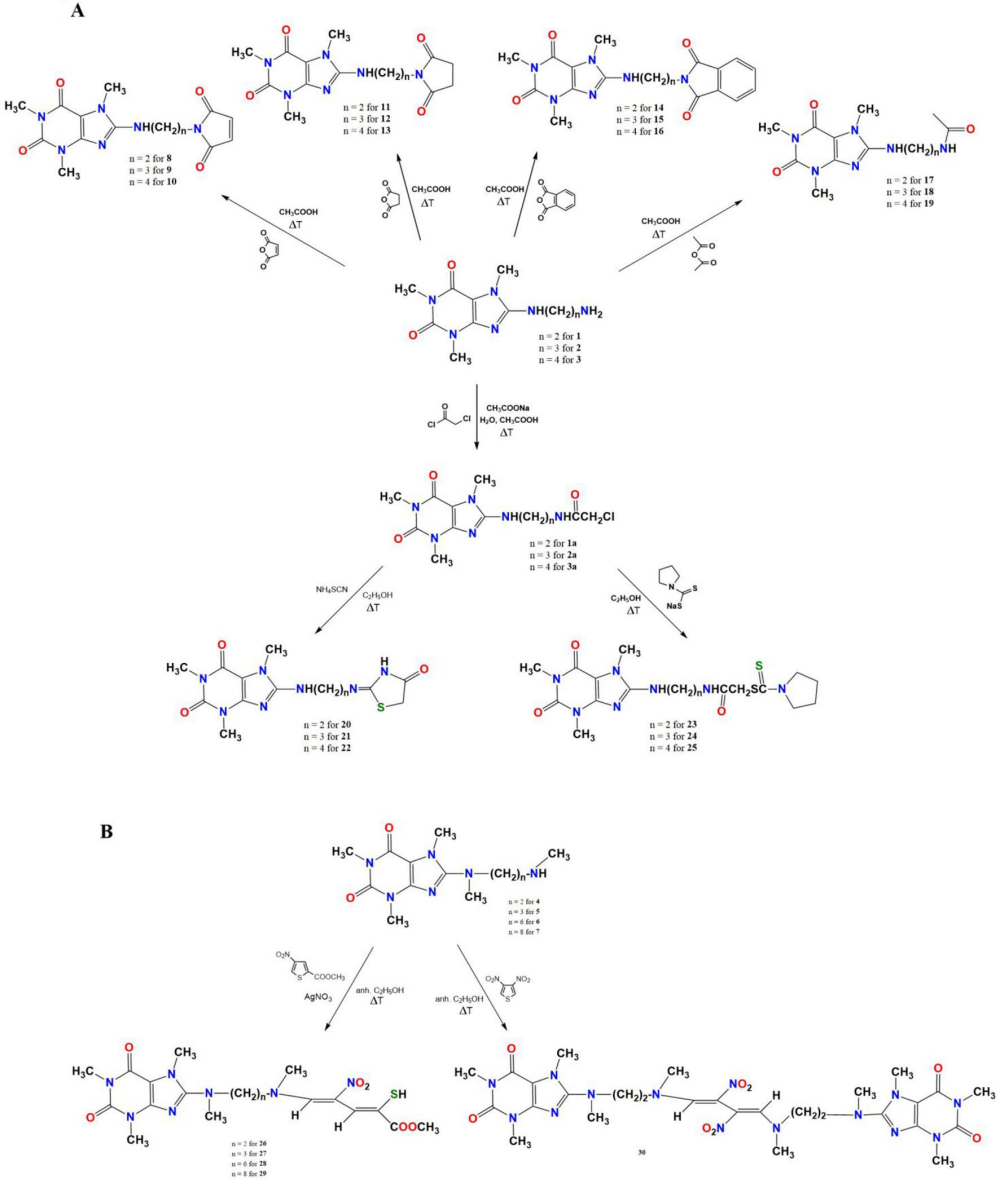
Synthesis of caffeine derivatives **2–25** (**A**) and **26–30** (**B**).

In our previous study^19^, we reported that selected di- and polyamine caffeine derivatives exhibit impressive chelating activity and inhibitory effect on hemolysis of human erythrocytes induced by AAPH-derived free radicals. In the present work, we present the synthesis of a new structurally diverse group of C8-substituted caffeine derivatives, obtained by modifying of selected diamine caffeine derivatives (**1**–**7**) by adding (i) amide or imide groups, (ii) thiazolidinones or pyrrolidinedithiocarbamates moieties, or (iii) alkadiene substituents (Fig. 1). The first group of compounds was obtained by nucleophilic *N*-substitution of caffeine derivatives **1–3** with anhydride moiety (maleic, succinic, phthalic, acetic) (Fig. 1A). The acetic anhydrides react with the primary amine group to give imide and amide caffeine derivatives (compounds **8–16** and **17–19**, respectively). The reactions were carried out in an acetic acid solution. Alternatively, compounds **1–3** in reaction with chloroacetyl chloride produced *N*-substituted acetamide **1a–3a** (Fig. 1A). This acylation reaction was carried out in an aqueous acetic acid solution with sodium acetate as a catalyst. Compounds **1a**–**3a** were intermediate products in synthesizing the second group of caffeine derivatives. The heterocyclization of compounds **1a–3a** in the presence of ammonium thiocyanate in refluxing ethanol produced 4-thiazolidinones **20–22.** Compounds **20–22** were formed through intramolecular cyclization and Dimroth-like rearrangements^35^. The *N*-substitution reaction of compounds **1a–3a** by sodium pyrrolidinedithiocarbamate in refluxing ethanol led to obtain compounds **23–25** (Fig. 1A). The third group of compounds was obtained by a ring-opening reaction of thiophene’s derivatives (Fig. 1B).

It is known that nitrothiophenes underwent a ring-opening reaction with secondary amines lead to potential pharmacologically active compounds^36,37^. The caffeine analogs **4**–**7** with terminal secondary amine group in reaction with asymmetric methyl 4-nitrothiophene-2-carboxylate gave mercaptans **26–29**. The reaction was carried out in the presence of AgNO_3_ in absolute ethanol. In turn, the reaction between compound **4** and symmetric 3,4-dinitrothiophene (3,4-DNT) in absolute ethanol at room temperature produced 1,4-disubstituted 2,3-dinitro-1,3-butadienes analogs **30**. Methyl 4-nitrothiophene-2-carboxylate is commercially available, and 3,4-dinitrothiophene was obtained by a three-step reaction according to^38^. First, 2,5-dibromothiophene was nitrated to 2,5-dibromo-3,4-dinitrothiophene. Next, the substituted thiophene and hydroiodic acid were mixed in acetone at room temperature to give 2-bromo-3,4-dinitrothiophene reacted with copper in refluxing acetic acid to give 3,4-dinitrothiophene.

The chemical structures of the final products **8**–**30** were assigned based on spectral data analyses (data available from the Supplementary).

### Iron chelating activity

**Figure 2:**
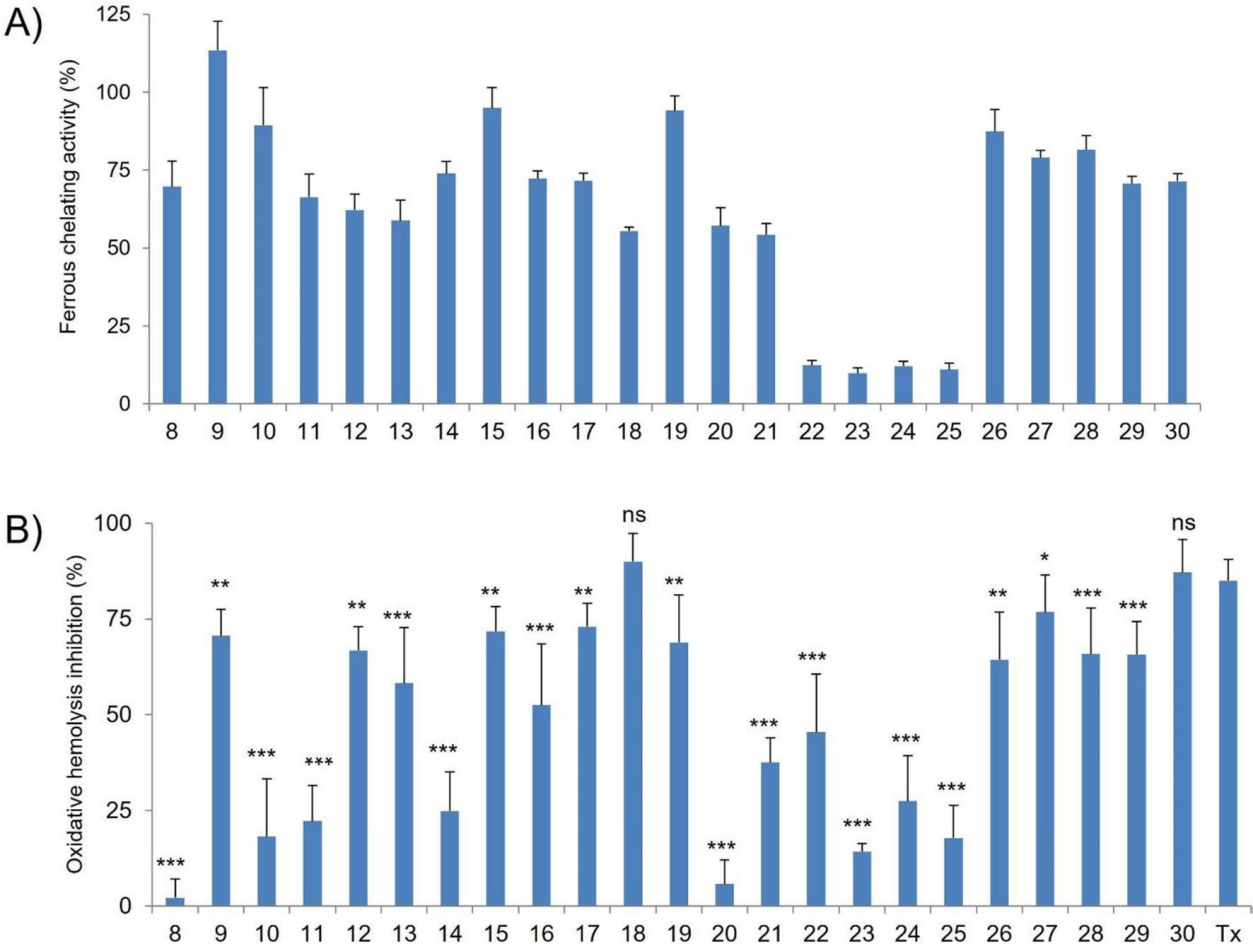
(**A**) The ferrous chelating activity of caffeine derivatives at 0.1 mg/mL presented as % activity of the standard chelator EDTA. The results are presented as mean value ± standard deviation. (**B**) In vitro protective activity of caffeine derivatives and standard antioxidant Trolox (Tx) at concentration of 0.1 mg/mL against AAPH-induced oxidative hemolysis. RBC were pre-incubated (20 min) with compounds and incubated (240 min) with 60 mM AAPH. The results are presented as the mean value ± standard deviation in comparison with Tx activity (*P < 0.05, **P < 0.01, ***P < 0.001). Non statistically significant difference is indicated as ns.

The metal chelating activity of antioxidants is important because it can reduce elevated concentration of iron, which is involved in the peroxidation of cellular components. In addition, metal chelation is a medical procedure to reduce the toxicity of metal by binding and excreting them from the body with effective chelating agents^14–17^. It has been shown that compounds containing two or more functional groups –OH, –COOH, –SH, –OCH_3_, –C=O, –PO_3_H_2_, –NR_2_, –O– and –S–, in the appropriate configuration, can bind ferrous ions^39^. Interactions of the caffeine with metal ions can occur through its oxygen and nitrogen atoms. However, due to the presence of methyl groups on N1, N3 and N7 atoms, caffeine forms complexes with metal ions through its O2 and O6 atoms^18^, and its chelating activity is low, namely 6%^18^ or 11%^19^, respectively compared to EDTA (100%), used as a reference chelator of ferrous ions (Fe^2+^). In this study, a ferrozine-based colorimetric assay was applied to investigate whether structural modification of C8-diaminoalkyl caffeine derivatives increases their chelating activity. As shown in Fig. 2A, all the new caffeine derivatives chelate Fe^2+^ in a structure-dependent manner, and some of them have high chelating efficiencies ranging from 54 to 113% of EDTA activity, respectively.

For the imide and amide derivatives of caffeine (Fig. 1, path a), the highest chelating activity was found for derivatives **9, 10**, **15,** and **19** (from 89 to 113% of EDTA activity), which depends on (i) the type of anhydride used and (ii) the length of the alkyl chain. The chelating properties of these derivatives can be attributed to the presence of carbonyl groups conjugated with a cis carbon–carbon double bond (**9**, **10**, **15**) or an amide group (**19**). The O, C and N atoms of the mentioned derivatives can form conjugated systems—two tautomeric (keto-enol) structures for **9**,**10**, and **15** and two resonance structures for compound **19**. Furthermore, the chelating activity can be increased with the length of the alkyl diamine chain.

Thiazolidinone can form metal complexes using nitrogen and oxygen as donor atoms^40^. The chelating properties of derivatives with a thiazolidinone group (compounds **20–22**) are variable (57, 54, and 12%, respectively). The lowest chelating activity of compound **22** (alkyl chain n = 4) indicates that two nitrogen atoms are involved in the metal coordination, one from the thiazolidinone molecule and one from the *N*-alkyl linker. The chelating efficiency of the derivatives containing a pyrrolidinedithiocarbamate moiety (compounds **23**–**25**), ranging from 9 to 12%, is similar to the chelating efficiency of caffeine (11%, see^19^). In this case, the low chelating activity is probably due to the unfavorable arrangement of the xanthine rings and the introduced moiety. There were no differences in Fe^2+^ chelating activity between the derivatives **26**–**30** (from 71 to 87%) and the starting compounds **4–7** (see^19^). The additional structural modification of starting compound molecules, especially in the case of compound **30**, which consists of two caffeine molecules, does not increase their iron chelating activity.

### Biocompatibility of derivatives

Carelli-Alinovi et al. showed that caffeine effectively protects the RBC membrane, including phospholipid asymmetry and band 3 protein function, against amyloid beta-peptide (1–42) induced oxidative alteration^41^. This excellent study supports the hypothesis of a protective role of caffeine in patients with Alzheimer’s disease (AD) and other neurodegenerative pathologies associated with oxidative stress. Caffeine is preferentially located in the hydrophobic region of the lipid bilayer of the cell membrane but cannot spontaneously partition from the aqueous environment^42^. On the other hand, the very high bioavailability of caffeine has been determined by in vivo studies^43^, so caffeine is an important model molecule in pharmaceutical science. To investigate the cytoprotective activity of the new caffeine derivatives, their membrane-disrupting activity was first assessed in a hemolysis assay using human RBC. It should be noted that the hemolytic activity of compounds higher than 5% excludes them from further evaluation at a given concentration^19,32,44^. For all derivatives used at a concentration of 0.1 mg/mL, no hemolytic activity higher than 5% was observed, namely the range of their hemolytic activity was from 1.48% ± 2.35 to 3.07% ± 1.82. Moreover, there was no modifying effect of the derivatives on RBC shape. The results obtained allow us to conclude that the structural modification of the starting molecules (see Fig. 1) resulted in non-cytotoxic, biocompatible (hemocompatible) compounds, for further evaluation of their cytoprotective activity.

### The cytoprotective activity of derivatives against AAPH-induced oxidative hemolysis

The cytoprotective properties of all derivatives against oxidative damage in RBC induced by peroxyl radicals (ROO^·^) generated by the thermal decomposition of the hydrophilic 2,2′-azobis(2-methylpropionamidine) dihydrochloride (AAPH) were evaluated at a concentration of 0.1 mg/mL. This chosen concentration has previously been used to assess the properties of di- and polyamine analogs of caffeine^19^ and gramine derivatives^44^. The cytoprotective activity of the derivatives was compared with that of Trolox, used as a reference antioxidant. As shown in Fig. 2B, the derivatives protect RBC against peroxyl radicals (ROO^·^)-induced oxidative hemolysis in a structure-dependent manner. Of the 33compounds tested, 13 showed cytoprotective activity higher than 50% of the activity of Trolox. Moreover, there was no statistically significant difference between the cytoprotective activity of derivatives **18** and **30,** and Trolox. On the other hand, the activity of derivatives **8**, **20**, **23**, **24**–**25** was similar to that obtained for caffeine, equal to 12% (see^19^).

Caffeine has been described to neutralize various ROS in one-step or multi-step reactions, respectively^9^. Mechanisms that involve an one-step reaction are RAF (radical adduct formation), SET (single electron transfer), and HAT (hydrogen atom transfer). In contrast, PCET (proton coupled electron transfer) and SEPT (sequential electron proton transfer) are multi-step mechanisms. Devasagayam et al. showed that caffeine inhibits membrane lipid peroxidation induced by various ROS in the following order: of hydroxyl radical (·OH), singlet oxygen (^1^O_2_), and peroxyl radicals (ROO^·^), respectively^7^. In addition, ·OH scavenging activity of caffeine via a SET mechanism has been proposed^45^.

In our study, newly synthesized caffeine derivatives effectively scavenge peroxyl radicals generated from AAPH and protect RBC due to the presence of substituents at the C-8 position, which can be explained by HAT, SET or RAF mechanisms. Galano et al. showed that the presence of electron-donating groups, such as –NH–COCH_3_, decreases the efficiency of the SET mechanism, while the presence of electron-withdrawing groups, such as NO_2_, increases it. The RAF mechanism is characterized as an antioxidant with multiple bonds^46^. Derivatives **17–19** with an NH amide group are the most efficient hydrogen atom donating compounds capable of reacting efficiently with peroxyl radicals using HAT mechanism. The activity of compounds **26–30** is probably due to electron-rich unsaturated bonds, which allow them to react as electron donors for the formation of a radical or the addition of a peroxyl radical. Moreover, the presence of unsaturated conjugated bonds promotes dislocation of the radical. Due to the presence of unsaturated bonds, derivatives **26–30** can successfully neutralize the peroxyl radical using the RAF mechanism. In addition, in the case of derivatives **26–29**, due to the presence of electron-withdrawing groups, the RAF mechanism can compete with the SET mechanism. Moreover, compounds **17**–**19** and **26**–**29** are structurally similar to vitamin E (tocopherol), a well-known natural antioxidant. These derivatives and vitamin E have a bicyclic system with an external branched alkyl chain. The "polar head-non-polar tail" structure of the molecule facilitates its interaction with the lipid bilayer of the RBC membrane. We have previously shown that the interaction of the "polar head-non-polar tail" molecule with the lipid bilayer can increase the stability of the RBC membrane and consequently enhance RBC protection against ROS^44^. Therefore, the highest cytoprotective activity of compound **18** (structurally similar to vitamin E) can be explained by its interaction with the cell membrane due to (i) the appropriate alkyl chain length and by (ii) its direct ROS scavenging activity, respectively. The electron donating ability of compound **30** is likely supported by the presence of two caffeine molecules linked by an alkyl chain (see Fig. 1).

### Evaluation of intracellular ROS level and hemoglobin oxidation

**Figure 3:**
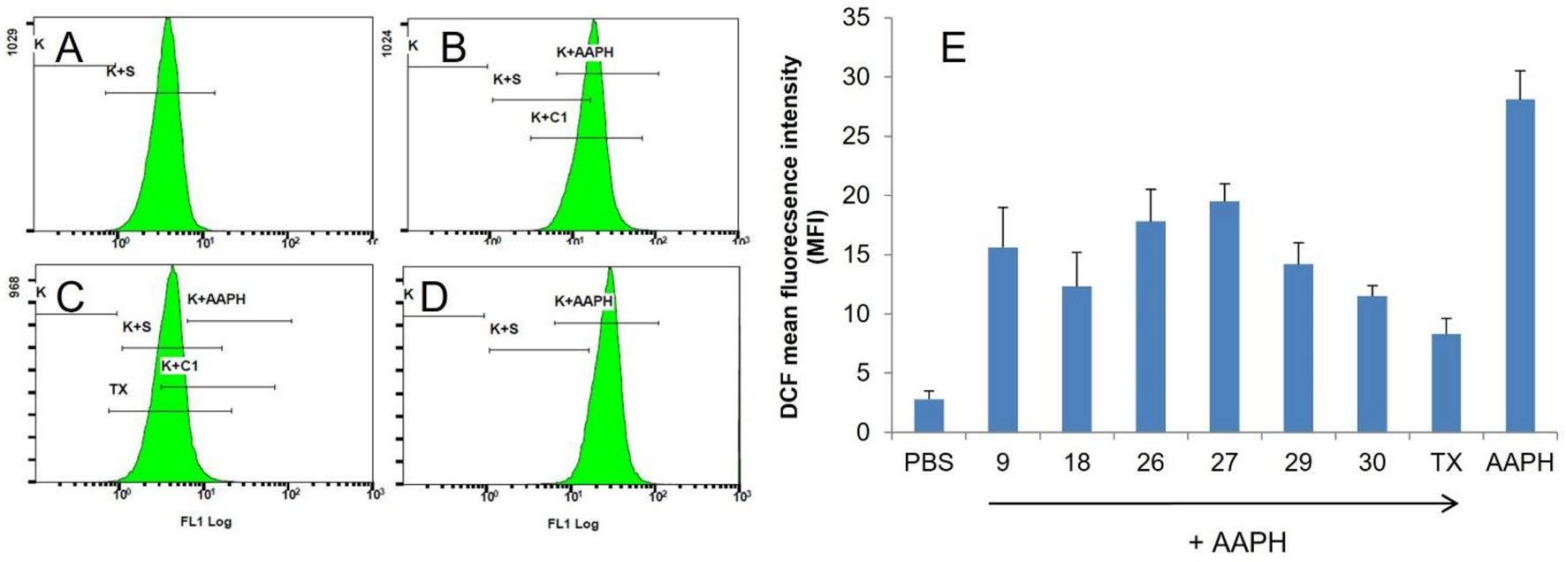
Inhibitory activity of selected caffeine derivatives on ROS level generated by 60 mM AAPH (1.5 h, 37 °C) in human RBC estimated as DCF mean fluorescence intensity (MFI). The representative flow cytometry histograms are presented: (**A**) PBS, negative control, (**B**) derivative 27 + AAPH, (**C**) Trolox, referenced antioxidant, + AAPH, (**D**) AAPH, positive control, (**E**) mean fluorescence intensity (MFI) values (± SD, n = 6) of DCF in RBC obtained for tested samples derivatives samples.

Selected derivatives with the highest cytoprotective activity (**9, 18, 26, 27, 29, 30**) were used to study their effects on AAPH-induced oxidative stress inside RBC in a cellular antioxidant assay (CAA)^47,48^. In this assay, the membrane-permeable hydrophobic fluorescent redox probe 2′,7′-dichlorodihydrofluorescein diacetate (DCF-DA) is deacetylated by cellular esterases and trapped inside RBC. DCF then undergoes ROS-dependent oxidation, leading to its conversion to a fluorescent form with fluorescence intensity dependent on ROS levels. Figure 3 shows histograms and presents mean fluorescence intensity (MFI) values obtained from the flow cytometer for the samples studied. As expected, the lowest level of ROS was detected in control RBC incubated in PBS buffer (Fig. 3A,E), and the highest in RBC incubated with AAPH (Fig. 3D,E). As shown in Fig. 3E, all selected derivatives reduced ROS levels within RBC in a structure-dependent manner, but the standard antioxidant Trolox was the most effective (also compare Fig. 3B with C). Based on the results, it can be concluded that all selected derivatives pass the RBC membrane and scavenge ROS inside RBC in a structure-dependent manner.

**Figure 4:**
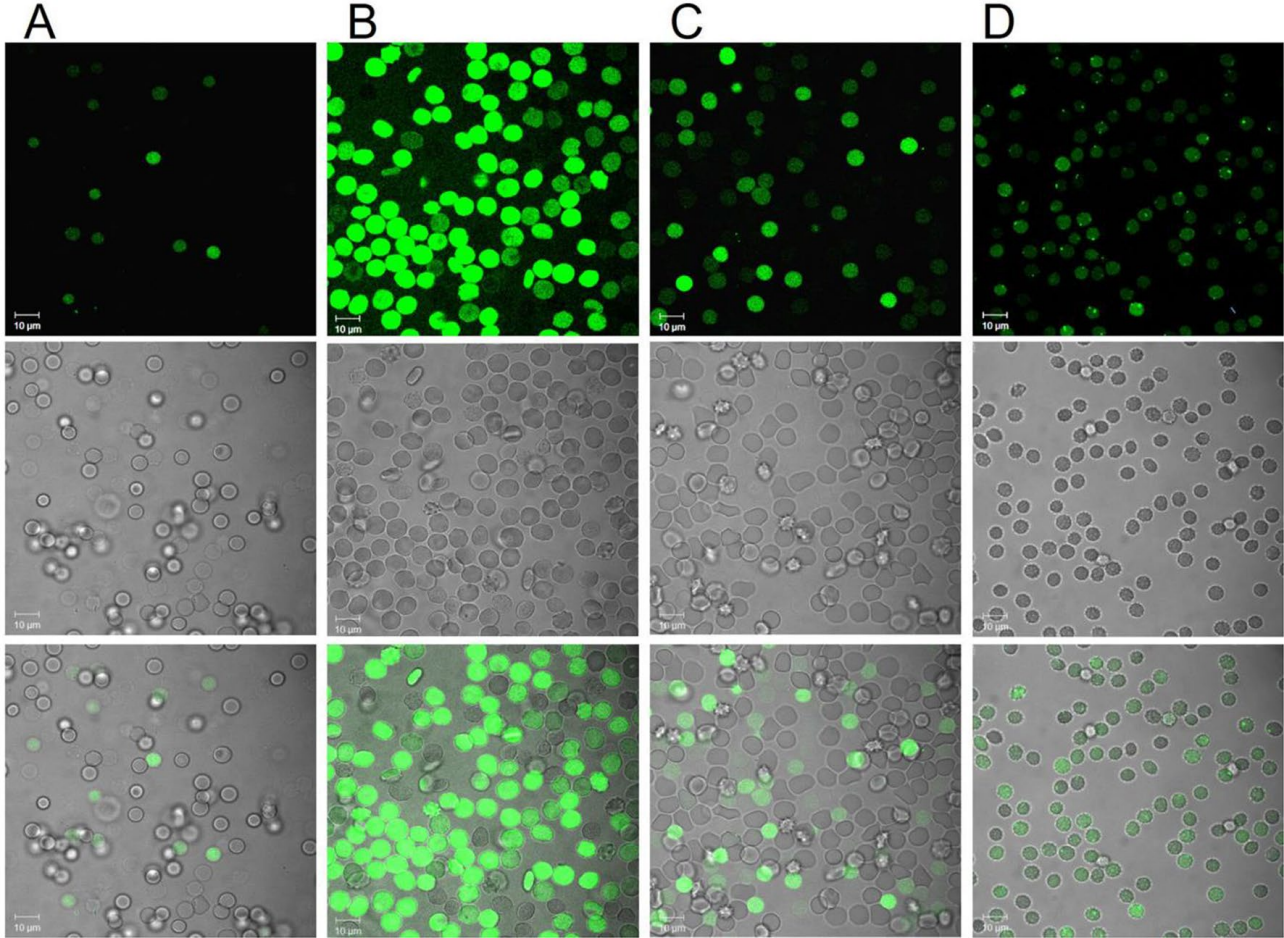
Supravital visualization of ROS level by fluorescent redox probe DCF-DA (10 µM, 30 min, 37 °C) in confocal microscopy: (**A**) control RBC (PBS), (**B**) 60 mM AAPH (2 h), (**C**) derivative 18 (0.1 mg/mL, 20 min preincubation) + 60 mM AAPH (1.5 h), (**D**) Trolox (0.1 mg/mL, 20 min preincubation) + 60 mM AAPH (1.5 h). The upper panel—fluorescence images, the middle panel—transmitted light images, the bottom panel—merge fluorescence and transmitted light images. The representative images of a series of experiments are presented. Scale bar = 10 µm.

Supravital confocal analysis of RBC was performed simultaneously with flow cytometry measurements. As expected, the fluorescent intensity of DCF-loaded RBC decreased significantly in the presence of derivative **18** (Fig. 4C) and the standard antioxidant Trolox (Fig. 4D) compared to the AAPH sample (Fig. 4B). The shape of RBC was mostly discocytic (Fig. 4A–C); however, echinocytes were observed in the presence of Trolox (Fig. 4D). It should be noted here that RBC are the most deformable cells in the human body to optimize blood flow properties in the circulatory system, and the stomatocyte–discocyte–echinocyte (SDE) transformation of RBC shape is a well-described phenomenon both in vivo^49^ and in vitro^50^. Therefore, the discocytic and echinocytic shapes of human RBC observed in this in vitro study are physiological.

**Figure 5:**
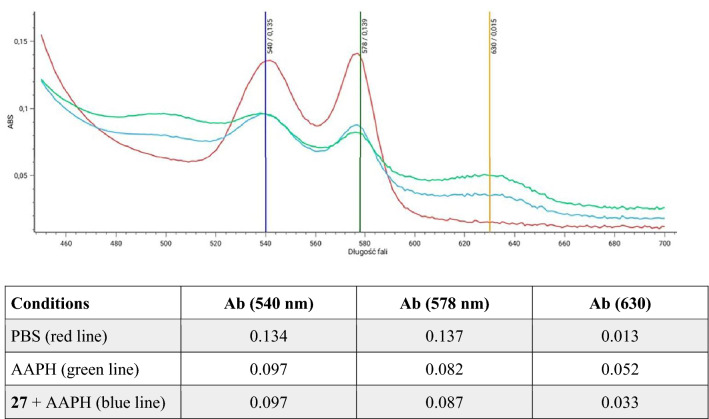
Spectral scans (450–700 nm) of hemoglobin (Hb) in supernatants after 4 h of incubation of RBC in PBS (red line), 60 mM AAPH (green line), derivative 27 (0.1 mg/mL) and 60 mM AAPH (blue line). Absorbance values (Ab) measured at 540, 578 (oxy-Hb peaks), and 630 nm (MetHb peak) are presented for every scan. The representative data for a series of experiments are presented.

Hemoglobin (Hb) is the main protein in RBC that performs the function of oxygen transport. Under physiological conditions, autooxidation of Hb continuously produces small amounts of methemoglobin (MetHb) with hem iron oxidized to iron(III)^51^. MetHb is converted back to Hb by nicotinamide adenine dinucleotide (NADH)-dependent cytochrome-b5 reductase. However, when ROS levels are elevated, the antioxidant system is ineffective, and oxygen transport to tissues is impaired leading to ischemia. To investigate the protective effect of selective derivatives against Hb oxidation, spectral scans of Hb (450–700 nm) were performed. In the control sample (PBS buffer), oxyhemoglobin is characterized by two peaks at 540 and 570 nm (Fig. 5, red line), and MetHb, which gives a peak at 630 nm, is absent. In the sample with AAPH, the oxy-Hb peak decreases and a peak specific to MetHb appears (Fig. 5, green line). In the presence of derivative **27**, there is no change in the oxy-Hb peaks compared to the AAPH sample, but the MethHb peak decreased (Fig. 5, blue line) from an absorbance value of 0.052 to 0.033 (see table in Fig. 5). In conclusion, derivative **27** protected Hb from MetHb formation (Fig. 5).

**Figure 6:**
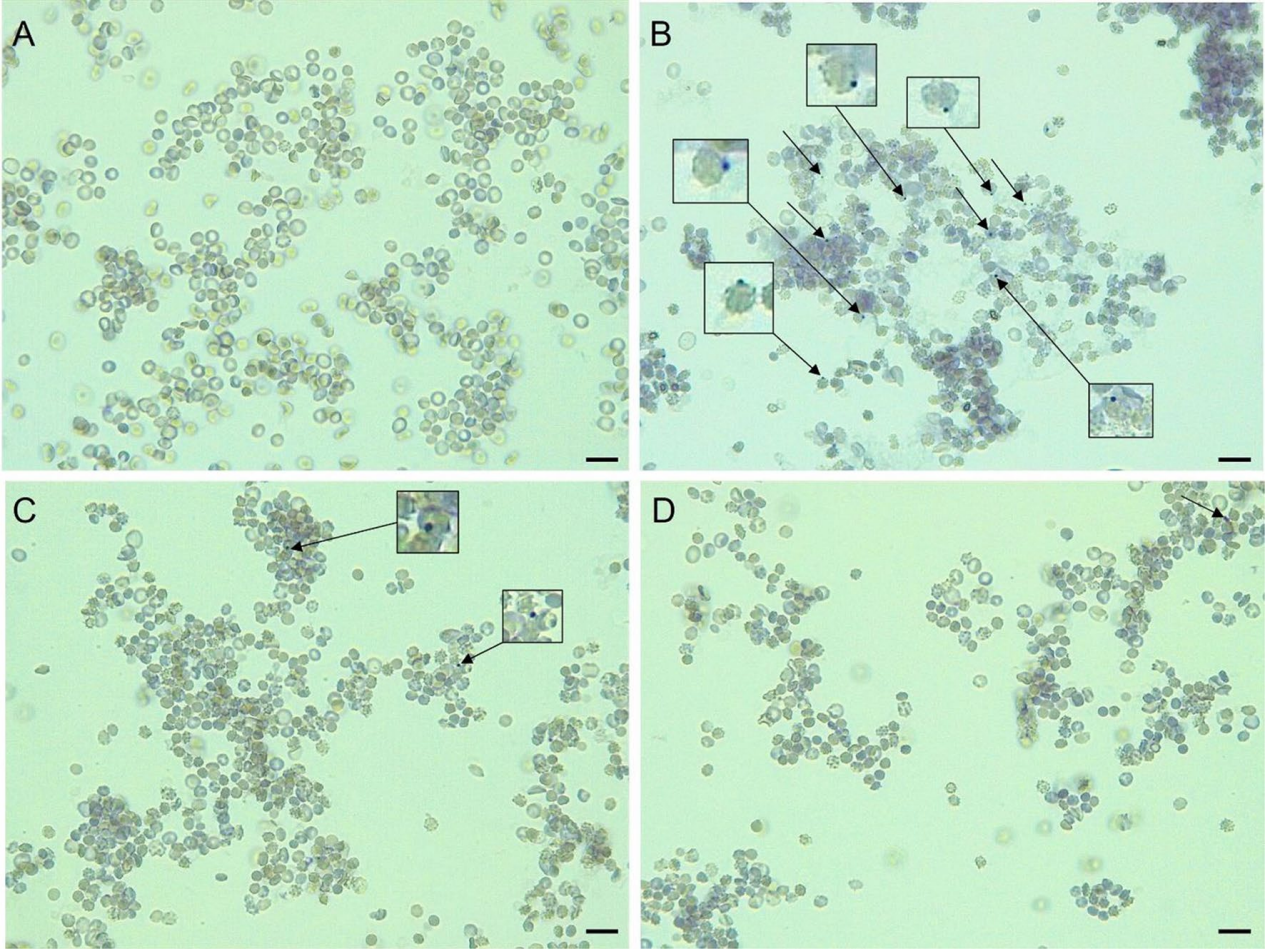
Supravital visualization of Heinz bodies in RBC stained with methyl violet (0.5%, 45 min, 37 °C) in light microscopy: (**A**) control RBC (PBS), (**B**) 60 mM AAPH (4 h); (**C**) derivative 27 (0.1 mg/mL, 20 min preincubation) + 60 mM AAPH (4 h); (**D**) Trolox (0.1 mg/mL, 20 min preincubation) + 60 mM AAPH (4 h). Inserts in (**B**) and (**C**) magnification of RBC with Heinz bodies visible as small, stained granules next to the RBC membrane. The representative images for a series of experiments are presented. Scale bar = 10 µm.

Heinz bodies are RBC inclusions of hemichrome formed from oxidized or denatured Hb, most commonly in old RBC or in certain medical conditions, including glucose-6-phosphate dehydrogenase (G-6-PD) deficiency and unstable Hb diseases^52^. Antioxidants with the ability to pass the cell membrane, such as the natural phenolic compound allylpyrocatechol, can significantly reduce the formation of Heinz bodies in RBC incubated with AAPH-derived free radicals^53^. Light microscopy analysis showed that selected derivatives, namely derivative **27,** reduced the Heinz bodies formation under AAPH-induced oxidative stress (Fig. 6). The inhibition of Hb oxidation by derivative **27** confirms its high hemoprotective efficacy.

In summary, our results obtained using different assays showed that selected C-8 substituted caffeine derivatives are: (i) effective ferrous ions chelators, (ii) biocompatible and cell membrane-passing compounds that (iii) effectively protect the RBC membrane and hemoglobin from ROS-induced damage.

### In silico evaluation of 8-substituted caffeine derivatives

In silico methods are one of the most effective tools for verifying the potential of a compounds as a drug and provide an opportunity to predict the ADME profile (absorption, distribution, metabolism, and excretion). Physicochemical properties of all compounds studied were calculated using the SwissADME web server.

As can be seen from Table 1, all the newly obtained compounds are more lipophilic than caffeine. Most of the new caffeine derivatives with a log P values between 1 and 3 have good lipophilicity allowing them to cross the lipid bilayer of the cell membrane. The highest log P values are observed in compounds containing an unsaturated alkyl substituent at C-8 of the xanthine moiety (compounds **26**–**29**); in general, the longer the alkyl side chain, the higher the partition coefficient. Compound **29** is the most lipophilic (log P 4.36) of all compounds tested. Furthermore, most of the compounds meet the criteria of "the rule of 5" (less than 5 HB donors, 10 HB acceptors, molecular weight < 500, and calculated log P < 5^54^), making them promising antioxidants. As mentioned before, the caffeine molecule preferentially locates in the hydrophobic part of the cell membrane^42^. Therefore, derivative **30,** having two caffeine molecules, may interact more efficiently with the hydrophobic part of the lipid bilayer, and, as a result, stabilize the molecular membrane structure against free radicals damage. In addition, compounds **26**–**30,** as hydrogen bond acceptors, can effectively interact with the cell membrane components through hydrogen bond and can prevent or delay ROS-RBC membrane interactions. Despite the well-known fact that caffeine is the most widely consumed central-nervous-system stimulant^55^, its selected C8-substituted derivatives may act as effective antioxidants and cytoprotective agents in prevention or treatment of neurodegenerative diseases.

**Table 1:** Physicochemical properties of 8-substituted aminocaffeine **8–30**.

Comp	Molecular weight	Num. of hydrogen bond acceptors	Num. of hydrogen bond donors	Num. of rotable bonds	Molecular polar surface area	LogP	Lipinski rules
Caffeine	194.19	3	0	0	61.82	1.79	Yes
**8**	332.31	5	1	4	111.23	2.18	Yes
**9**	346.34	5	1	5	111.23	2.39	Yes
**10**	360.37	5	1	6	111.23	2.66	Yes
**11**	334.33	5	1	4	111.23	2.28	Yes
**12**	348.36	5	1	5	111.23	2.47	Yes
**13**	362.38	5	1	6	111.23	2.68	Yes
**14**	382.37	5	1	4	111.23	2.62	Yes
**15**	396.40	5	1	5	111.23	2.90	Yes
**16**	410.43	5	1	6	111.23	3.12	Yes
**17**	294.31	4	2	5	102.95	1.86	Yes
**18**	308.34	4	2	6	102.95	2.43	Yes
**19**	322.36	4	2	7	102.95	2.41	Yes
**20**	351.38	5	2	4	140.61	2.17	Yes
**21**	365.41	5	2	5	140.61	2.18	Yes
**22**	379.44	5	2	6	140.61	2.69	Yes
**23**	439.56	4	2	9	163.58	3.00	Yes
**24**	453.58	4	2	10	163.58	2.98	Yes
**25**	467.61	4	2	11	163.58	3.43	Yes
**26**	467.50	7	0	9	179.22	3.18	Yes
**27**	481.53	7	0	10	179.22	3.52	Yes
**28**	523.61	7	0	13	179.22	4.28	No
**29**	551.66	7	0	15	179.22	4.36	No
**30**	700.71	10	0	13	228.24	4.05	No

## Conclusions

Caffeine is one of the well-known and most commonly consumed psychoactive compound. Daily consumption of caffeine may be part of a diet that protects against cognitive decline and dementia that progresses with age. Therefore, the subject of our study was to investigate the effect of structural modifications of caffeine on its antioxidant and cytoprotective efficacy in human erythrocytes as model cells.

Our results showed a significant relationship between the structure of biocompatible C8-substituted caffeine derivatives and their ability to (i) complex ferrous ions and (ii) protect human RBC from the harmful effects of oxidative stress. The highest chelating activity of compounds **9**, **10**, **15**, and **19** can be attributed to the presence of tautomeric or resonances structures with an -OH group, which enables them to form complexes with ferrous ions. A mechanism of SET, RAF and/or HAT and radical stabilization has been proposed to explain the antioxidant potential of the derivatives. Derivatives **17–19** with NH amide group, and derivatives **26**–**30**, bearing unsaturated conjugated bonds that promote radical relocation, have shown high protective activity in human erythrocytes. Derivatives **17**–**19** neutralize the peroxyl radical via the HAT mechanism, while derivatives **26**–**30** neutralize via the RAF mechanism. Moreover, in case of derivatives **26–29**, the RAF mechanism can compete with the SET mechanism. In addition, the high cytoprotective activity of derivatives **18** and **30** may be related to their specific amphiphilic structure which allows them to interact with the hydrophobic and/or hydrophilic region of the cell membrane through hydrogen bonds, respectively. The interaction of the derivatives with the cell membrane results in its stabilization, which protects both membrane components and hemoglobin from ROS-induced oxidation.

In summary, our results obtained both in vitro and in silico indicate that iron chelation and the specific interaction with the human erythrocyte membrane are responsible for the impressive cytoprotective properties of selected C-8 substituted caffeine derivatives. Since most of the newly obtained biocompatible caffeine derivatives fulfill the criteria Lipinski’s rule of five, they can be used for further structural modification to extend our knowledge of the structure–activity relationships of caffeine-based cytoprotective compounds with beneficial pharmacological properties.

## Materials and methods

### Instrumentation and chemicals: a general experimental procedure

All starting materials were obtained from Sigma-Aldrich and were used without purification. Proton (^1^H) and carbon (^13^C) NMR spectra were recorded with a Varian Gemini 300/400 spectrometer operating at 300 and 75 MHz with DMSO-*d*_*6*_ as the solvent and TMS as the internal standard. Chemical shifts are reported in *δ* (parts per million). FTIR spectra were recorded on a Nicolet iS5 Spectrometer (KBr pellets). EI mass spectra were measured on a 320-MS/450 GC mass spectrometer (Bruker). Melting points were determined with an SMP-20 apparatus (BŰCHI Labortechnik AG). Analytical thin-layer chromatography (TLC) was carried out on silica gel plates 60 F254 (Sigma-Aldrich). Detection on TLC was made by UV light.

### Synthesis of 8-substituted aminocaffeine

The synthesis of compounds **1–7** was described in our previous paper^19^.

### A typical procedure for the reaction of diaminocaffeine analogs with acetic anhydrides (compounds 8–19)

A mixture of proper 8-diaminocaffeine derivatives **1–3** (1 mmol) and the appropriate anhydride (1 mmol) in acetic acid (10 mL) was heated under reflux for 1–15 h and cooled to room temperature. The obtained mixture was extracted with CH_2_Cl_2_ and dried over MgSO_4_. The solution was evaporated, then crude product **8–19** was recrystallized from CHCl_3_.

### A typical procedure for the reaction of diaminocaffeine analogs with chloroacetic chloride (compounds 1a–3a)

To a solution of appropriate diaminocaffeine analogs, **1–3** (1 mmol) in acetic acid (97% aqueous solution; 10 mL) was added dropwise to a solution of 2-chloroacetyl chloride (97% aqueous solution, 0.08 mL, 1 mmol). When the reaction mixture reached a temperature of 40 °C, sodium acetate (320 mg, 1 mmol) in distilled water (8 mL) was added. The reaction mixture was stirred for 8 h at a temperature of 40–60 °C. The reaction mixture was cooled to room temperature and extracted by CHCl_3_ (3 × 20 mL). The combined organic layer was dried over MgSO_4_ and evaporated under reduced pressure to give an oil.

### A typical procedure for the synthesis of compounds 20–22

A mixture of appropriate caffeine derivatives (**1a**, **2a,** or **3a**) (1 mmol) and ammonium thiocyanate (76.12 mg, 1 mmol for **1a**; 152.24 mg, 2 mmol for **2a** and **3a**) in ethanol (10 mL) was heated under reflux for 22–43 h, and when cooled to room temperature. The solution was evaporated to give an oil.

### A typical procedure for the synthesis of compounds 23–25

A mixture of appropriate caffeine derivatives (**1a**, **2a**, or **3a**) (1 mmol) and sodium pyrrolidinedithiocarbamate (338.48 mg, 2 mmol) in ethanol (10 mL) was heated under reflux for 33–40 h, and when cooled to room temperature. The solution was evaporated to give an oil.

### Typical procedure for the ring opening of methyl 4-nitrothiophene-2-carboxylate (compounds 26–29)

A suspension of methyl 4-nitrothiophene-2-carboxylate (1 mmol; 187 mg) in anhydrous ethanol (5 mL) with magnetic stirring was heated to 40 °C, and silver nitrate (1 mmol; 170 mg) was added. After 15 min, the appropriate caffeine diamine derivatives **4**–**7** (1 mmol) were introduced, the reaction mixture was heated under reflux for 20 h and kept for three days under magnetic stirring. It was then cooled to room temperature and diluted with water (10 mL). The water phase was extracted by CH_2_Cl_2_ (3 × 30 mL) and dried over MgSO_4_. The organic solvent was evaporated under reduced pressure to give a crude product, which was crystallized from petroleum ether.

### Synthesis of 8,8′-[N,N,N′,N′-(1,4,9,12-tetramethyl)-5,7-dienyl-6,7-dinitro-1,4,9,12-tetraaza-dodecano]-dicaffeine

A suspension of 3,4-dinitrothiophene (1 mmol; 174 mg) in anhydrous ethanol (5 mL) with magnetic stirring was heated to 40 °C. After 15 min, 8-(methyl(2-(methylamino)ethyl)amino)caffeine (2 mmol; 568 mg) was introduced, the reaction mixture was heated under reflux for 20 h and kept for 3 days under magnetic stirring. The reaction mixture was cooled to room temperature and diluted with water (10 mL). The water phase was extracted by CH_2_Cl_2_ (3 × 30 mL) and dried over MgSO_4_. The organic solvent was evaporated under reduced pressure to give a residue, and the crude product was crystallized from petroleum ether.

### Ferrous ions (Fe^2+^) chelating activity assay

Ferrous ions (Fe^2+^) chelating activity was evaluated by inhibition the formation of the Fe^2+^-ferrozine complex after incubation of the tested compounds with Fe^2+^. The Fe^2+^-chelating ability of the tested compounds was determined by the absorbance of the ferrous ion-ferrozine complex at 562 nm at room temperature (~ 22 °C, RT). In brief, 0.1 mg/mL concentration of the compounds tested in 0.2 mL ethyl alcohol was added to a solution of 0.6 mM FeCl_2_ (0.05 mL). EDTA was used as the standard metal chelator. The reaction was started by adding 5 mM ferrozine (0.05 mL) in ethyl alcohol and shaking vigorously immediately. The samples were stored for 10 min at room temperature. Following incubation, the absorbance (Abs) of the solutions was measured at 562 nm in a BioMate™ 160 UV–Vis spectrophotometer. The percentage of inhibition of ferrozine–Fe^2+^ complex formation was calculated using the equation:

1 $${\text{Fe}}^{{{2} + }} \;{\text{chelating}}\;{\text{ effect }}\left( \% \right) \, = \, [{1} - ({\text{Abs}}_{{1}} /{\text{Abs}}_{0} )] \, \times { 1}00$$

where Abs_0_ is the absorbance of the sample without the tested compound and Abs_1_ is the absorbance in the presence of the compound tested. Each sample was made in triplicate and three independent experiments were performed.

### Human RBC preparation

All methods were carried out in accordance with the relevant guidelines and regulations, and the Bioethics Committee approved all experimental protocols for Scientific Research at the Medical University of Poznań (agreement no. ZP/907/1002/18). Human red blood cell concentrates were purchased from Blood Bank in Poznań without any contact with blood donors. Informed consent was obtained from all blood donors.

Freshly human RBC suspensions (hematocrit 65%) were washed three times (3000 rpm, 10 min, + 4 °C) in 7.4 pH phosphate buffered saline (PBS—137 mM NaCl, 2.7 mM KCl, 10 mM Na_2_HPO_4_, 1.76 mM KH_2_PO_4_) supplemented with 10 mM glucose. After washing, cells were suspended in PBS buffer at 1.65 × 10^9^ cells/mL (hematocrit 15%), stored at 4 °C, and used within 5 h.

### Hemolytic assay

Hemolytic activity was evaluated as previously reported^19^. Briefly, RBC (1.65 × 10^8^ cells/mL, hematocrit 1.5%) were incubated in PBS (7.4 pH) supplemented with 10 mM glucose and containing compounds tested at a concentration equal to 0.1 mg/mL for 60 min at 37 °C in shaking incubator. The concentration of derivatives used in this study was selected according to our previous research^19,44^. Samples with RBC incubated in PBS without compounds tested were taken as the control sample. Each sample was repeated three times, and the experiments were repeated four times with RBC from different donors. After incubation, RBC suspensions were centrifuged (3000 rpm, 10 min, + 4 °C), and the degree of hemolysis was estimated by measuring the absorbance of the supernatant at λ = 540 nm in a BioMate™ 160 UV–Vis spectrophotometer. The results were expressed as a percentage (%) of hemolysis. Hemolysis 0% was taken as the absorbance of the supernatant of RBC suspensions in PBS buffer, while the total hemolysis (100%) was determined when PBS was replaced by ice-cold distilled water. Hemolysis degree < 5% indicates no hemolytic activity of a compound.

### Microscope evaluation of human RBC shape

Following incubation with compounds at a concentration of 0.1 mg/mL, RBC were fixed in 5% paraformaldehyde (PFA) plus 0.01% glutaraldehyde (GA) for one hour at RT. Fixed RBC were washed by exchanging supernatant with PBS. After washing, RBC were settled on poly-l-lysine-treated (0.1 mg/mL, 10 min, at RT) cover glasses and mounted on 80% glycerol. The cover slips were sealed with nail polish. A large number of cells in several separate experimental samples were studied using a Zeiss LSM 510 (AXIOVERT ZOOM) confocal microscope (Carl Zeiss Microscopy, Oberkochen, Germany) (100 ×/1.4 aperture immersion oil objective, 10 × ocular). Images were acquired using the Zeiss LSM Image Browser program (Carl Zeiss Microscopy, Oberkochen, Germany).

### Inhibition of oxidative stress-induced hemolysis

The cytoprotective activity of derivatives was evaluated according to the previously described method^44^. Briefly, RBC (1.65 × 10^8^ cells/mL, 1.5% hematocrit) were preincubated in PBS (pH 7.4) supplemented with 10 mM glucose, containing compound tested or Trolox used as the standard antioxidant at a concentration of 0.1 mg/mL for 20 min at 37 °C in the shaking incubator. The concentration of derivatives was selected according to our previous studies^19,44^. After pre-incubation, 2,2′-azobis(2-methylpropionamidine) dihydrochloride (AAPH) was added at a final concentration of 60 mM, and samples were incubated for the next four hours. Erythrocytes incubated in PBS, and incubated with AAPH, were taken as the negative and positive controls, respectively. After incubation, the RBC suspensions were centrifuged (3000 rpm, 5 min, + 4 °C), and the degree of hemolysis was determined by measuring the absorbance (Abs) of the supernatant at λ = 540 nm in BioMate™ 160 UV–Vis spectrophotometer. The percentage of ROS-induced hemolysis inhibition was calculated using the following equation:

2 $${\text{Inhibition }}\;{\text{of }}\;{\text{hemolysis }}\;\left( \% \right) \, = { 1}00{-}[({\text{Abs}}_{{{\text{comp}}}} {-}{\text{Abs}}_{{{\text{PBS}}}} /{\text{Abs}}_{{{\text{AAPH}}}} {-}{\text{Abs}}_{{{\text{PBS}}}} ) \, \times { 1}00\% ]$$

where Abs_comp_ is the absorbance value of supernatant obtained from samples incubated with a compound tested in the presence of AAPH, Abs_PBS_ is the absorbance of the supernatant obtained from negative control, and Abs_AAPH_ is the absorbance of the supernatant obtained from positive control, respectively. Each sample was made in triplicate, and the results are presented as a mean value (± SD) of three independent experiments with RBC obtained from different donors.

### Evaluation of ROS level inside RBC using flow cytometry

ROS level was detected as previously reported^48^. Briefly, redox fluorescent probe 2’,7’-dichlorodihydrofluorescein diacetate (DCF-DA) (Sigma-Aldrich D6883) was used to estimate the ROS level in intact RBC. RBC preincubated with selected derivatives or Trolox (as described above) and incubated with 60 mM AAPH (1.5 h, 37 °C), were washed (3000 rpm, 5 min, + 4 °C) and then incubated with DCF-DA at a final concentration of 10 μM at 37 °C for 30 min in the dark. DCFDA-loaded RBC (100 µl) were resuspended in 1000 μL PBS, and immediately, ROS-dependent DCF-fluorescence intensity in RBC was measured at an excitation wavelength of 488 nm and an emission wavelength of 530 nm on a flow cytometer Cytomix FC 500 MPL (Beckman Coulter). The results were presented as histograms and the values of mean fluorescence intensity (MFI).

### Visualization of ROS inside RBC in the confocal microscope

RBC prepared for flow cytometry analysis (as above) were washed (3000 rpm, 5 min, + 4 °C) and settled on poly-l-lysine-treated (0.1 mg/mL, 10 min, RT) cover glasses and mounted on 80% glycerol. Immediately, living cells were analyzed using a Zeiss LSM 510 (AXIOVERT ZOOM) confocal microscope (Carl Zeiss Microscopy, Oberkochen, Germany), (100 ×/1.4 aperture immersion oil objective, 10 × ocular) at an excitation wavelength of 488 nm and an emission wavelength of 530 nm. Images were acquired using the Zeiss LSM Image Browser program (Carl Zeiss Microscopy, Oberkochen, Germany).

### Methemoglobin detection

The absorption spectra of hemoglobin were scanned between the visible range from 450 to 700 nm in BioMate™ 160 UV–Vis spectrophotometer, using supernatants obtained from selected samples obtained in the section “Inhibition of oxidative stress-induced hemolysis”. RBC obtained from selected samples in the section “Inhibition of oxidative stress-induced hemolysis” were used for intracellular visualization of Heinz bodies. Cells were stained with methyl violet (0.5% in 0.9% NaCl) for 45 min at RT. Following incubation, RBC were washed and fixed in 5% PFA plus 0.01% GA for one hour at RT. Fixed RBC were washed by exchanging supernatant with PBS. After washing, RBC were settled on poly-l-lysine-treated (0.1 mg/mL, 10 min, RT) cover glasses and mounted on 80% glycerol. The cover slips were sealed with nail polish. A large number of cells in several separate experimental samples were studied using a RED-233 MOTIC microscope (63 x/1.4 aperture, 10 × ocular). Images were acquired using the Motic Images Plus 3.0.

### In silico evaluation of C-8 substituted aminocaffeine

The physicochemical properties of all compounds tested were calculated using the SwissADME web server: www.swissadme.ch.

### Statistical analysis

For antioxidant and cytoprotective properties, data were plotted as the mean ± SD of three or four independent experiments with every sample in triplicate (n = 9). A paired *t*-Student test was used to compare the derivatives activity with the activity of the standard antioxidant Trolox. Statistical significance was defined as *P* < 0.05. No statistically significant difference was indicated as ns.

## Supplementary Information

Supplementary Information.

## Data Availability

The datasets used and/or analyzed during the current study available from the corresponding author on reasonable request.
