# Supplementary material for: New C8-substituted caffeine derivatives as promising antioxidants and cytoprotective agents in human erythrocytes
Source: Sci Rep. 2023 Jan 31;13:1785. doi: 10.1038/s41598-022-27205-8 (PMC9888743; doi:10.1038/s41598-022-27205-8)
Supplement: Supplementary file 1 — Supplementary Information. [file 41598_2022_27205_MOESM1_ESM.docx]

**Supplementary data**

**New C8-substituted caffeine derivatives as promising antioxidants and cytoprotective agents in human erythrocytes**

Arleta Sierakowska^1^, Beata Jasiewicz^1*^, Łukasz Piosik^2^†, Lucyna Mrówczyńska*^2^

^1^Department of Bioactive Products, Faculty of Chemistry, Adam Mickiewicz University in Poznań, Uniwersytetu Poznańskiego 8, 61-614 Poznań, Poland; *E-mail: [beatakoz@amu.edu.pl](mailto:beatakoz@amu.edu.pl) (BJ)

^2^Department of Cell Biology, Faculty of Biology, Adam Mickiewicz University in Poznań, Uniwersytetu Poznańskiego 6, 61-614 Poznań, Poland; *E-mail: [lumro@amu.edu.pl](mailto:lumro@amu.edu.pl) (LM)

† - In memoriam, 18.08.2021

**TABLE OF CONTENTS**

Spectroscopic study of caffeine derivatives…………….……………………...…………………...S3

^1^H NMR spectrum of 8-((2-maleimidethyl)amino)caffeine **8**…………………..…………..………S9

^1^H and ^13^C NMR spectra of 8-((4-maleimidbutyl)amino)caffeine **10**…....….….…………………S10

^1^H and ^13^C NMR spectra of 8-(2-(pyrrolidine-2,5-dion)ethylamino)caffeine **11**………….…...…S11

^1^H NMR spectrum of 8-(4-(pyrrolodine-2,5-dion)butylamino)caffeine **13**……………………….S12

^1^H and ^13^C NMR spectra of 8-((2-phthalimidethyl)amino)caffeine **14**…………………………...S13

^1^H NMR spectrum of 8-((4-phthalimidbutyl)amino)caffeine **16**………………..………………...S14

^1^H and ^13^C NMR spectra of 8-(2-aminoethyl)-N-acetylaminocaffeine **17**……………..……....…S15

^1^H NMR spectrum of 8-(3-aminopropyl)-N-acetylaminocaffeine **18**.........……..………………...S16

^1^H and ^13^C NMR spectra of 8-{N-[(tiazylidyn-4-on)-2^’^-imino]ethyloamino}caffeine **20**……….S17

^1^H and ^13^C NMR spectra of 8-{N-[(tiazylidyn-4-on)-3^’^-imino]propyloamino}-caffeine **21** ...…S18

^1^H and ^13^C NMR spectra of 8-{N-[acetylo-(pirolid-1-ylokarbotionylosulfanylo)]-aminobutylo-amino}caffeine **23**………….…....….….......……..…......………...…......................……..……..S19

^1^H and ^13^C NMR spectra of 8-{N-[acetylo-(pirolid-1-ylokarbotionylosulfanylo)]-aminobutylo-amino]caffeine **25………………………………………………………………….…………**.…...S20

^1^H NMR and ^13^C NMR spectra of 8-[N,N’-(1,4-dimethyl)-1,4-diaza-5,7-dienyl-8-mercapto-8-metoxycarbonyl-6-nitro-octano]caffeine **26**…………...…..……………….…….……....……….S21

^1^H NMR and ^13^C NMR spectra of 8-[N,N’-(1,5-dimethyl)-1,5-diaza-6,8-dienyl-9-mercapto-9-metoxycarbonyl-7-nitro-nonano]caffeine **27**………….…......…..…......….…..….....……...…….S22

^1^H and ^13^C NMR spectra of 8-[N,N’-(1,8-dimethyl)-1,8-diaza-9,11-dienyl-12-mercapto-12-metoxycarbonyl-10-nitro-dodecano]caffeine **28**…………………………………………………..S23

^1^H and ^13^C NMR spectra of 8-[N,N’-(1,10-dimethyl)-1,10-diaza-11,13-dienyl-14-mercapto-14-metoxycarbonyl-12-nitro-tetradecano]caffeine **29**……...…………………………………………S24

^1^H and ^13^C NMR spectra of 8,8’-[N,N,N’,N’-(1,4,9,12-tetramethyl)-5,7-dienyl-6,7-dinitro- 1,4,9,12tetraazadodecano]dicaffeine **30…**…………………………………………………….......S25

***8-{N-[chloroacethyl]aminoethyloamino]}caffeine (1a)***

Oil (157 mg, 48 %); ^1^H NMR (300 MHz, DMSO-*d_6_*): δ 7.20 (s, 1H), 4.06 (s, 2H), 3.91 (s, 1H), 3.54 (s, 3H), 3.41-3.38 (m, 4H), 3.34 (s, 3H), 3.16 (s, 3H); ^13^C NMR (75 MHz, DMSO-*d_6_*): 166.15, 153.94, 152.86, 150.93, 148.22, 101.88, 42.65, 42.58, 41.65, 38.50, 29.68, 27.12; FT-IR: (KBr): 3306, 1703, 1698, 751; EI MS (m/z, % int.): 328 (M^+^, 15 %).

***8-{N-[chloroacethyl]aminopropylamino]}caffeine (2a)***

Oil (120 mg, 35 %); ^1^H NMR (300 MHz, DMSO-*d_6_*): δ 7.11 (s, 1H), 4.07 (s, 1H), 3.83 (s, 2H), 3.56 (s, 3H), 3.41-3.37 (m, 2H), 3.34 (s, 3H), 3.16 (s, 3H), 3.14-3.08 (m, 2H), 2.87-2.82 (m, 2H); ^13^C NMR (75 MHz, DMSO-*d_6_*): 165.91, 154.52, 152.86, 150.96, 148.35, 101.82, 42.61, 36.62, 36.46, 35.97, 29.27, 28.92, 27.13; FT-IR: (KBr): 3306, 1703, 1698, 751; EI MS (m/z, % int.): 342 (M^+^, 80 %).

***8-{N-[chloroacethyl]aminobutylamino]}caffeine (3a)***

Oil (93 mg, 26 %); ^1^H NMR (300 MHz, DMSO-*d_6_*): δ 7.02 (s, 1H), 4.13 (s, 2H), 4.04 (s, 1H), 3.55 (s, 3H), 3.34 (s, 3H), 3.16 (s, 3H), 3.14-3.03 (m, 4H), 1.61-1.56 (m, 2H), 1.43-1.38 (m, 2H); ^13^C NMR (75 MHz, DMSO-*d_6_*): 165.73, 154.13, 152.83, 150.95, 148.37, 101.77, 42.65, 42.03, 41.61, 38.65, 29.70, 29.22, 27.11, 26.56; FT-IR: (KBr): 3306, 1703, 1698, 751; EI MS (m/z, % int.): 356 (M^+^, 48 %).

***8-((2-maleimidethyl)amino)caffeine (8)***

Solid (242 mg, 73 %); mp: 220-222 ^0^C; ^1^H NMR (300 MHz, DMSO-*d_6_*): δ 6.98 (s, 2H), 4.55 (s, 1H), 3.63-3.60 (m, 2H), 3.56-3.51 (m, 2H), 3.46 (s, 3H), 3.30 (s, 3H), 3.16 (s, 3H); FT-IR: (KBr): 3430, 1710, 1654, 1615; EI MS (m/z, % int.): 332 (M^+^, 100 %).

***8-((3-maleimidpropyl)amino)caffeine (9)***

Solid (201 mg, 58 %) mp: 180-182 ^0^C; ^1^H NMR (300 MHz, DMSO-*d_6_*): δ 7.01 (s, 2H), 4.66 (s, 1H), 3.56-3.54 (m, 2H), 3.51 (s, 3H), 3.42-3.39 (m, 2H), 3.35 (s, 3H), 3.17 (s, 3H), 2.09-2.08 (m, 2H); FT-IR: (KBr): 3430, 1710, 1654, 1615; EI MS (m/z, % int.): 346 (M^+^, 2 %).

***8-((4-maleimidbutyl)amino)caffeine (10)***

Solid (216 mg, 60 %) mp: 210-212 ^0^C; ^1^H NMR (300 MHz, DMSO-*d_6_*): δ 7.01 (s, 2H), 4.57 (s, 1H), 3.56-3.54 (m, 2H), 3.51 (s, 3H), 3.42-3.37 (m, 2H), 3.35 (s, 3H), 3.17 (s, 3H), 2.03-1.99 (m, 2H), 1.79-1.77 (m, 2H); ^13^C NMR (75 MHz, DMSO-*d_6_*): 171.03, 168.90, 154.12, 152.83, 150.93, 148.36, 134.41, 129.52, 101.77, 69.74, 59.22, 57.14, 42.09, 37.94, 29.67, 27.10; FT-IR: (KBr): 3430, 1710, 1654, 1615; EI MS (m/z, % int.): 360 (M^+^, 32 %).

***8-(2-(pyrrolidine-2,5-dion)ethylamino)caffeine (11)***

Solid (147 mg, 44 %) mp: 200-202 ^0^C; ^1^H NMR (300 MHz, DMSO-*d_6_*): δ 3.55 (s, 1H), 3.52 (s, 3H), 3.41-3.37 (m, 2H), 3.34 (s, 3H), 3.17 (s, 3H), 2.58-2.55 (m, 6H); ^13^C NMR (75 MHz, DMSO-*d_6_*): 177.67, 169.41, 153.83, 152.85, 150.92, 148.25, 101.85, 37.83, 35.95, 35.88, 29.19, 27.97, 27.82, 27.09; FT-IR: (KBr): 3430, 1710, 1654; EI MS (m/z, % int.): 334 (M^+^, 80 %).

***8-(3-(pyrrolodine-2,5-dion)propylamino)caffeine (12)***

Solid (247 mg, 71 %) mp: 270-272^0^C; ^1^H NMR (300 MHz, DMSO-*d_6_*): δ 3.55 (s, 1H), 3.51 (s, 3H), 3.41-3.39 (m, 2H), 3.34 (s, 3H), 3.17 (s, 3H), 2.61-2.59 (m, 4H), 2.58-2.56 (m, 2H), 2.45-2.42 (m, 2H); FT-IR: (KBr): 3430, 1710, 1654; EI MS (m/z, % int.): 348 (M^+^, 100 %).

***8-(4-(pyrrolodine-2,5-dion)butylamino)caffeine (13)***

Solid (304 mg, 84 %) mp: 230-232 ^0^C; ^1^H NMR (300 MHz, DMSO-*d_6_*): δ 3.55 (s, 1H), 3.51 (s, 3H), 3.42-3.39 (m, 2H), 3.35 (s, 3H), 3.17 (s, 3H), 3.02-2.97 (m, 4H), 2.56–2.53 (m, 2H), 1.46-1.41 (m, 4H); FT-IR: (KBr): 3430, 1710, 1654; EI MS (m/z, % int.): 362 (M^+^, 100 %).

***8-((2-phthalimidethyl)amino)caffeine (14)***

Solid (317 mg, 83 %) mp: 160-162 ^0^C; ^1^H NMR (300 MHz, DMSO-*d_6_*): δ 7.88-7.77 (m, 4H), 3.85 (s, 3H), 3.63-3.60 (m, 2H), 3.54 (s, 1H), 3.45 (s, 3H), 3.30-3.26 (m, 2H) 3.12 (s, 3H); ^13^C NMR (75 MHz, DMSO-*d_6_*): 169.41, 167.92, 153.90, 152.69, 150.69, 147.94, 134.49, 134.20, 131.32, 123.08, 122.89, 122.75, 101.67, 37.56, 36.79, 36.28, 29.55, 27.05; FT-IR: (KBr): 3430, 3050, 1710, 1654; EI MS (m/z, % int.): 382 (M^+^, 40 %).

***8-((3-phthalimidpropyl)amino)caffeine (15)***

Solid (368 mg, 93 %) mp: 150-152 ^0^C; ^1^H NMR (300 MHz, DMSO-*d_6_*): δ 7.88-7.82 (m, 4H), 3.70-3.67 (m, 2H), 3.59 (s, 1H), 3.55 (s, 3H), 3.42-3.39 (m, 2H), 3.35 (s, 3H), 3.17 (s, 3H), 3.11-3.02 (m, 2H); FT-IR: (KBr): 3430, 3050, 1710, 1654; EI MS (m/z, % int.): 396 (M^+^, 8 %).

***8-((4-phthalimidbutyl)amino)caffeine (16)***

Solid (303 mg, 74 %) mp: 160-162 ^0^C; ^1^H NMR (300 MHz, DMSO-*d_6_*): δ 7.88-7.82 (m, 4H), 3.62 (s, 1H), 3.60-3.57 (m, 2H), 3.51 (s, 3H), 3.42-3.39 (m, 2H), 3.35 (s, 3H), 3.15 (s, 3H), 3.05-3.00 (m, 2H), 2.68-2.65 (m, 2H); FT-IR: (KBr): 3430, 3050, 1710, 1654; EI MS (m/z, % int.): 410 (M^+^, 4 %).

***8-(2-aminoethyl)-N-acetylaminocaffeine (17)***

Solid (244 mg, 83 %) mp: 160-162 ^0^C; ^1^H NMR (300 MHz, DMSO-*d_6_*): δ 7.08 (s, 1H), 3.55 (s, 3H), 3.35 (s, 3H), 3.17 (s, 3H), 3.06-3.04 (m, 4H), 1.80 (s, 1H), 1.79 (s, 3H); ^3^C NMR (300 MHz, DMSO-*d_6_*): 169.24, 153.94, 152.86, 150.93, 148.27, 101.86, 42.03, 29.67, 29.23, 27.13, 26.43, 22.60; FT-IR: (KBr): 3430, 1710, 1654; EI MS (m/z, % int.): 294 (M^+^, 11 %).

***8-(3-aminopropyl)-N-acetylaminocaffeine (18)***

Solid (83 mg, 27 %) mp: 200-202 ^0^C; ^1^H NMR (300 MHz, DMSO-*d_6_*): δ 6.96 (s, 1H), 3.55 (s, 3H), 3.35 (s, 3H), 3.17 (s, 3H), 3.13-2.99 (m, 2H), 1.80 (s, 1H), 1.79 (s, 3H), 1.73-1.66 (m, 2H), 1.53-1.46 (m, 2H); FT-IR: (KBr): 3430, 1710, 1654; EI MS (m/z, % int.): 308 (M^+^, 60 %).

***8-(4-aminobutyl)-N-acetylaminocaffeine (19)***

Solid (97 mg, 30 %) mp: 198-200 ^0^C; ^1^H NMR (300 MHz, DMSO-*d_6_*): δ 6.99 (s, 1H), 3.55 (s, 3H), 3.35 (s, 3H), 3.17 (s, 3H), 3.10-2.99 (m, 2H), 1.84 (s, 1H), 1.78 (s, 3H), 1.59-1.51 (m, 2H), 1.48-1.41 (m, 2H), 1.38-1.33 (m, 2H); FT-IR: (KBr): 3430, 1710, 1654; EI MS (m/z, % int.): 322 (M^+^, 100 %).

***8-{N-[(tiazylidyn-4-on)-2^’^-imino]ethyloamino}caffeine (20)***

Oil (298 mg, 85 %); ^1^H NMR (300 MHz, DMSO-*d_6_*): δ 8.09 (s, 1H), 4.54 (s, 1H), 3.91 (s, 2H), 3.62–3.60 (m, 2H), 3.55 (s, 3H), 3.34 (s, 3H), 3.27-3.21 (m, 2H), 3.17 (s, 3H); ^13^C NMR (75 MHz, DMSO-*d_6_*): 169.90, 153.98, 152.89, 150.97, 148.31, 129.51, 101.90, 42.06, 38.56, 38.46, 36.54, 29.32, 27.21; FT-IR: (KBr): 3080, 1708, 1696, 750; EI MS (m/z, % int.): 351 (M^+^, 4 %).

***8-{N-[(tiazylidyn-4-on)-2^’^-imino]propyloamino}caffeine (21)***

Oil (346 mg, 95 %); ^1^H NMR (300 MHz, Oil DMSO-*d_6_*): δ 8.08 (s, 1H), 4.54 (s, 1H), 3.92 (s, 2H), 3.58 (s, 3H), 3.52–3.50 (m, 2H), 3.34 (s, 3H), 3.16 (s, 3H), 3.11-3.09 (m, 2H), 2.91-2.88 (m, 2H); ^13^C NMR (75 MHz, DMSO-*d_6_*): 168.70, 154.05, 152.85, 150.95, 148.23, 129.74, 101.85, 44.24, 41.80, 36.58, 36.04, 29.80, 29.31, 27.16; FT-IR: (KBr): 3080, 1708, 1696, 750; EI MS (m/z, % int.): 365 (M^+^, 16 %).

***8-{N-[(tiazylidyn-4-on)-2^’^-imino]butyloamino}caffeine (22)***

Oil (379 mg, 97 %); ^1^H NMR (300 MHz, DMSO-*d_6_*): δ 8.09 (s, 1H), 4.28 (s, 1H), 3.94 (s, 2H), 3.64–3.62 (m, 4H), 3.56 (s, 3H), 3.34 (s, 3H), 3.16 (s, 3H), 1.63-1.55 (m, 2H), 1.50-1.39 (m, 2H);^13^C NMR (75 MHz, DMSO-*d_6_*): 169.00, 154.16, 152.83, 150.96, 148.40, 129.44, 101.79, 42.03, 41.61, 38.22, 36.29, 29.82, 29.28, 27.18, 26.63; FT-IR: (KBr): 3080, 1708, 1696, 750; EI MS (m/z, % int.): 379 (M^+^, 8 %).

***8-{N-[acetylo-(pirolid-1-ylokarbotionylosulfanylo)]aminoethyloamino}caffeine (23)***

Oil (417 mg, 95 %); ^1^H NMR (300 MHz, DMSO-*d_6_*): δ 7.35 (s, 1H), 4.01 (s, 1H), 3.75-3.71 (m, 2H), 3.67-3.63 (m, 2H), 3.62-3.58 (m, 2H), 3.57 (s, 3H), 3.41-3.39 (m, 2H), 3.35 (s, 3H), 3.17 (s, 3H), 2.98-2.95 (m, 2H), 2.05-2.00 (m, 2H), 1.93-1.87 (m, 2H); ^13^C NMR (75 MHz, DMSO-*d_6_*): 192.73, 166.83, 154.08, 152.83, 150.94, 148.29, 101.88, 55.18, 54.45, 50.22, 44.45, 44.20, 32.57, 29.29, 27.15, 25.65, 23.78; FT-IR: (KBr): 3347, 1701, 1695, 1033, 750; EI MS (m/z, % int.): 439 (M^+^, 4 %).

***8-{N-[acetylo-(pirolid-1-ylokarbotionylosulfanylo)]aminopropyloamino}caffeine (24)***

Oil (435 mg, 96 %); ^1^H NMR (300 MHz, DMSO-*d_6_*): δ 7.36 (s, 1H), 4.01 (s, 1H), 3.77-3.71 (m, 2H), 3.65-3.60 (m, 4H), 3.57 (s, 3H), 3.47-3.43 (m, 2H), 3.34 (s, 3H), 3.16 (s, 3H), 3.10-3.05 (m, 2H), 2.05-2.00 (m, 2H), 1.93-1.86 (m, 2H), 1.73-1.70 (m, 2H); ^13^C NMR (75 MHz, DMSO-*d_6_*): 192.83, 166.48, 154.12, 152.83, 150.97, 148.39, 101.83, 55.17, 54.44, 50.23, 44.38, 41.78, 36.64, 28.93, 27.15, 26.40, 25.66, 23.79; FT-IR: (KBr): 3347, 1701, 1695, 1033, 750; EI MS (m/z, % int.): 453 (M^+^, 4 %).

***8-{N-[acetylo-(pirolid-1-ylokarbotionylosulfanylo)]aminobutyloamino}caffeine (25)***

Oil (444 mg, 95 %); ^1^H NMR (300 MHz, DMSO-*d_6_*): δ 7.30 (s, 1H), 3.99 (s, 1H), 3.75-3.71 (m, 2H), 3.65-3.60 (m, 4H), 3.56 (s, 3H), 3.47-3.43 (m, 2H), 3.34 (s, 3H), 3.16 (s, 3H), 2.99-2.96 (m, 2H), 2.04-1.98 (m, 2H), 1.92-1.85 (m, 4H), 1.79-1.75 (m, 2H); ^13^C NMR (75 MHz, DMSO-*d_6_*): 192.77, 169.52, 154.21, 152.81, 150.96, 148.43, 101.78, 55.14, 54.45, 50.23, 44.27, 44.24, 38.23, 29.77, 29.27, 27.14, 26.56, 26.37, 24.20; FT-IR: (KBr): 3347, 1701, 1695, 1033, 750; EI MS (m/z, % int.): 467 (M^+^, 3 %).

***8-[N,N’-(1,4-dimethyl)-1,4-diaza-5,7-dienyl-8-mercapto-8-metoxycarbonyl-6-nitro-octano]-caffeine (26)***

Solid (363 mg, 77 %) mp: 110-112 ^0^C; ^1^H NMR (300 MHz, DMSO-*d_6_*): δ 9.05 (s, 1H), 8.18 (s, 1H), 8.07 (s, 1H), 3.89 (s, 9H), 3.52 (s, 3H), 3.33 (s, 6H), 3.21-3.18 (m, 2H), 2.69-2.65 (m, 2H); ^13^C NMR (75 MHz, DMSO-*d_6_*): 164.63, 160.53, 155.83, 150.62, 147.26, 135.04, 133.86, 129.50, 126.97, 111.25, 69.75 (x2), 62.94, 61.95, 52.94, 32.38, 28.95, 27.23; FT-IR: (KBr): 1722-1696, 1655, 1612, 1530; EI MS (m/z, % int.): 472 (M^+^, 32 %).

***8-[N,N’-(1,5-dimethyl)-1,5-diaza-6,8-dienyl-9-mercapto-9-metoxycarbonyl-7-nitro-nonano]-caffeine (27)***

Solid (185 mg, 38 %) mp: 120-122 ^0^C; ^1^H NMR (300 MHz, DMSO-*d_6_*): δ 9.07 (s, 1H), 8.20 (s, 1H), 8.07 (s, 1H), 3.89 (s, 9H), 3.51 (s, 6H), 3.34 (s, 3H), 3.20-3.16 (m, 2H), 2.67-2.65 (m, 2H), 1.91-1.83 (m, 2H); ^13^C NMR (75 MHz, DMSO-*d_6_*): 164.69, 160.53, 155.91, 149.15, 147.27, 135.05, 133.86, 129.52, 126.97, 108.94, 69.74 (x2), 62.96, 61.94, 52.94, 47.32, 35.15, 28.96, 21.77; FT-IR: (KBr): 1722-1696, 1655, 1612, 1530; EI MS (m/z, % int.): 487 (M^+^, 9 %).

***8-[N,N’-(1,8-dimethyl)-1,8-diaza-9,11-dienyl-12-mercapto-12-metoxycarbonyl-10-nitro-dodecano]caffeine (28)***

Solid (121 mg, 23 %) mp: 138-140 ^0^C; ^1^H NMR (300 MHz, DMSO-*d_6_*): δ 9.06 (s, 1H), 8.19 (s, 1H), 8.06 (s, 1H), 3.89 (s, 6H), 3.69 (s, 3H), 3.51 (s, 3H), 3.35 (s, 3H), 3.28-3.24 (m, 2H), 3.19 (s, 3H), 2.87-2.83 (m, 2H), 1.61-1.54 (m, 4H), 1.34-1.30 (m, 4H); ^13^C NMR (75 MHz, DMSO-*d_6_*): 160.57, 156.96, 153.49, 150.90, 147.28, 135.10, 133.88, 129.55, 127.00, 103.80, 69.77 (x2), 52.98, 52.64, 48.16, 32.62, 32.48, 29.29, 27.28, 26.56, 25.55, 25.29; FT-IR: (KBr): 1722-1696, 1655, 1612, 1530; EI MS (m/z, % int.): 528 (M^+^, 36 %).

***8-[N,N’-(1,10-dimethyl)-1,10-diaza-11,13-dienyl-14-mercapto-14-metoxycarbonyl-12-nitro-tetradecano]caffeine (29)***

Oil (222 mg, 40 %); ^1^H NMR (300 MHz, DMSO-*d_6_*): δ 9.06 (s, 1H), 8.19 (s, 1H), 8.06 (s, 1H), 3.89 (s, 6H), 3.69 (s, 3H), 3.51 (s, 3H), 3.35 (s, 3H), 3.28-3.21 (m, 2H), 3.18 (s, 3H), 2.87-2.83 (m, 2H), 1.61-1.54 (m, 6H), 1.34-1.30 (m, 6H); ^13^C NMR (75 MHz, DMSO-*d_6_*): 160.56, 156.98, 153.50, 150.90, 147.13, 135.08, 133.87, 129.54, 126.99, 103.78, 69.76 (x2), 52.96, 52.73, 48.41, 32.65, 29.26, 29.23, 28.51, 28.43, 27.27, 26.72, 25.97, 25.83; FT-IR: (KBr): 1722-1696, 1655, 1612, 1530; EI MS (m/z, % int.): 556 (M^+^, 79 %).

***8,8’-[N,N,N’,N’-(1,4,9,12-tetramethyl)-5,7-dienyl-6,7-dinitro-1,4,9,12-tetraazadodecano]-dicaffeine (30)***

Solid (280 mg, 40 %) mp: 40-42 ^0^C; ^1^H NMR (300 MHz, DMSO-*d_6_*): δ 8.39 (s, 2H), 3.67 (s, 6H), 3.32 (s, 6H), 3.17 (s, 6H), 3.04-2.99 (m, 8H), 2.82 (s, 12H). ^13^C NMR (75 MHz, DMSO-*d_6_*): 163.02, 162.76, 156.16, 156.10, 153.62, 153.51, 151.43, 150.87, 146.92, 146.69, 115.24 (x4), 104.11, 103.97, 57.02 (x2), 50.65 (x2), 48.91 (x2), 45.74 (x2), 35.40, 32.40, 29.37, 29.23, 27.35, 27.28; FT-IR: (KBr): 1722-1696, 1655, 1612, 1530; EI MS (m/z, % int.): 350 (½ M^+^, 10 %).

**
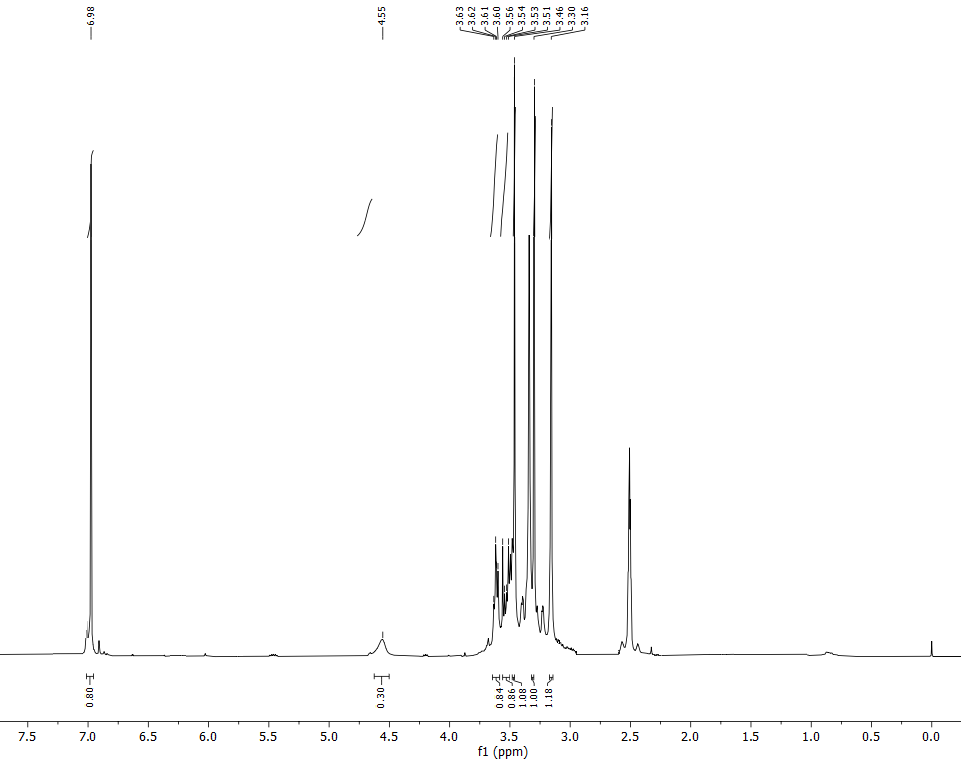
Figure S1a.** ^1^H NMR spectrum of 8-((2-maleimidethyl)amino)caffeine **8**.


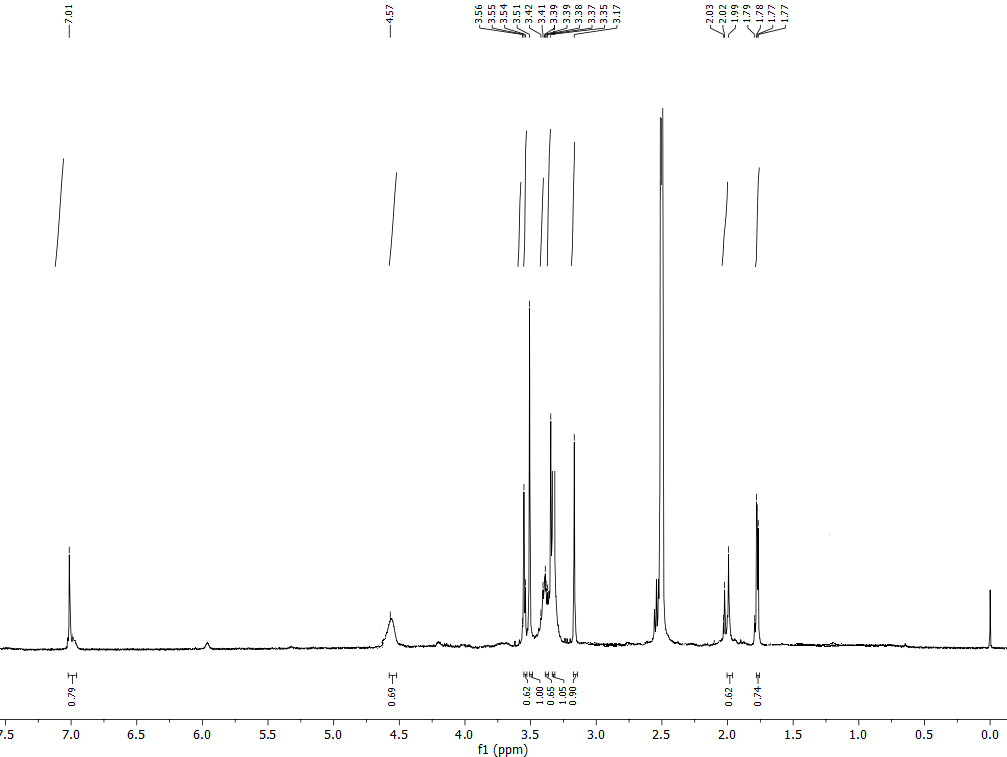
 **Figure S2a.** ^1^H NMR spectrum of 8-((4-maleimidbutyl)amino)caffeine **10**.


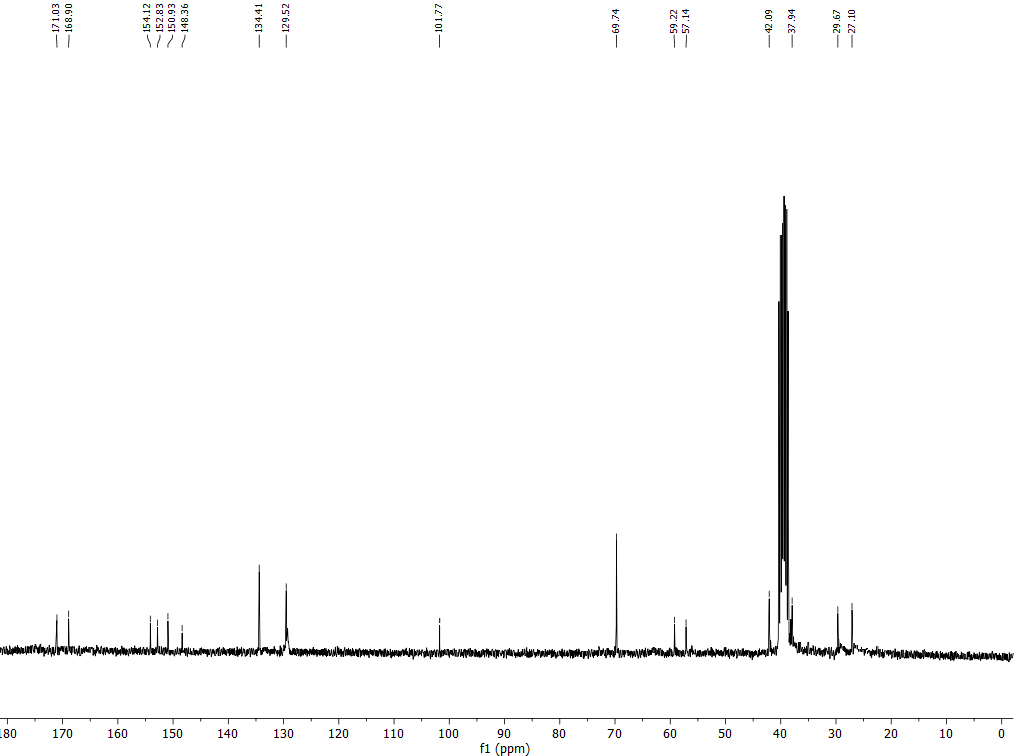
**Figure S2b.** ^13^C NMR spectrum of 8-((4-maleimidbutyl)amino)caffeine **10**.


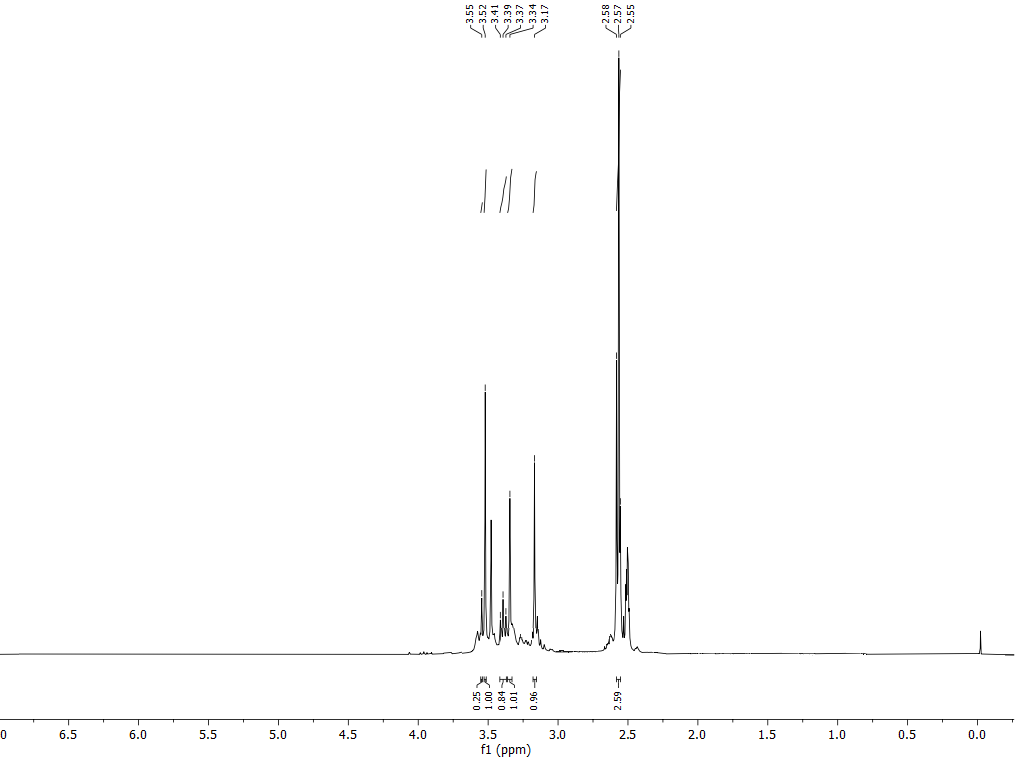
**Figure S3a.** ^1^H NMR spectrum of 8-(2-(pyrrolidine-2,5-dion)ethylamino)caffeine **11**.


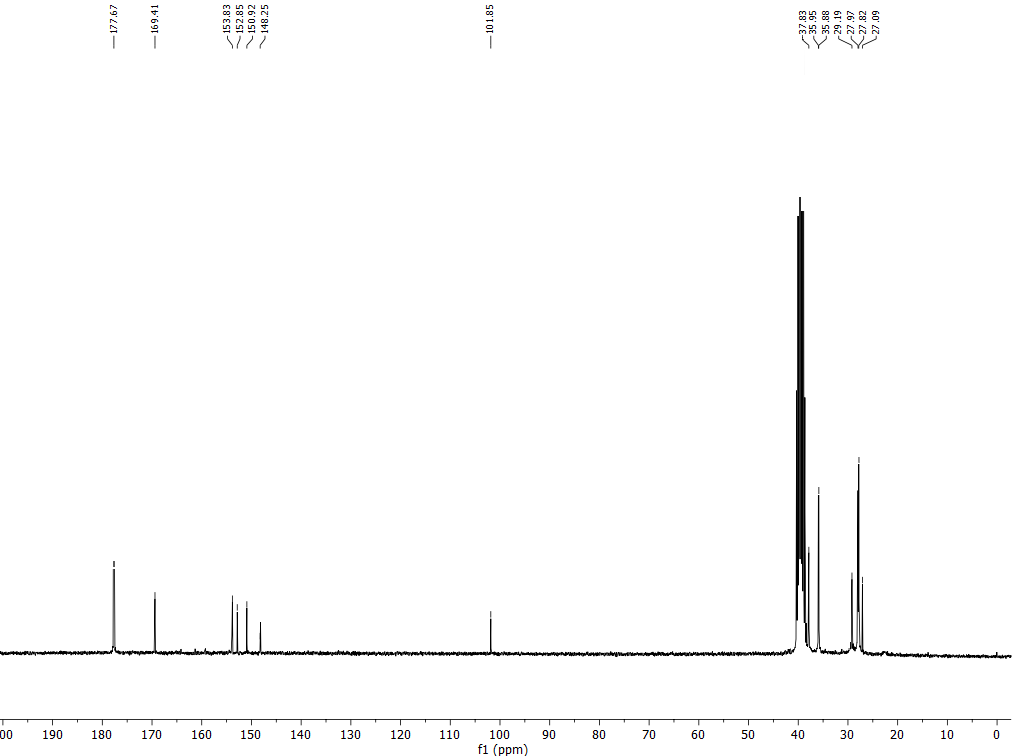
**Figure S3b.** ^13^C NMR spectrum of 8-(2-(pyrrolidine-2,5-dion)ethylamino)caffeine **11**.


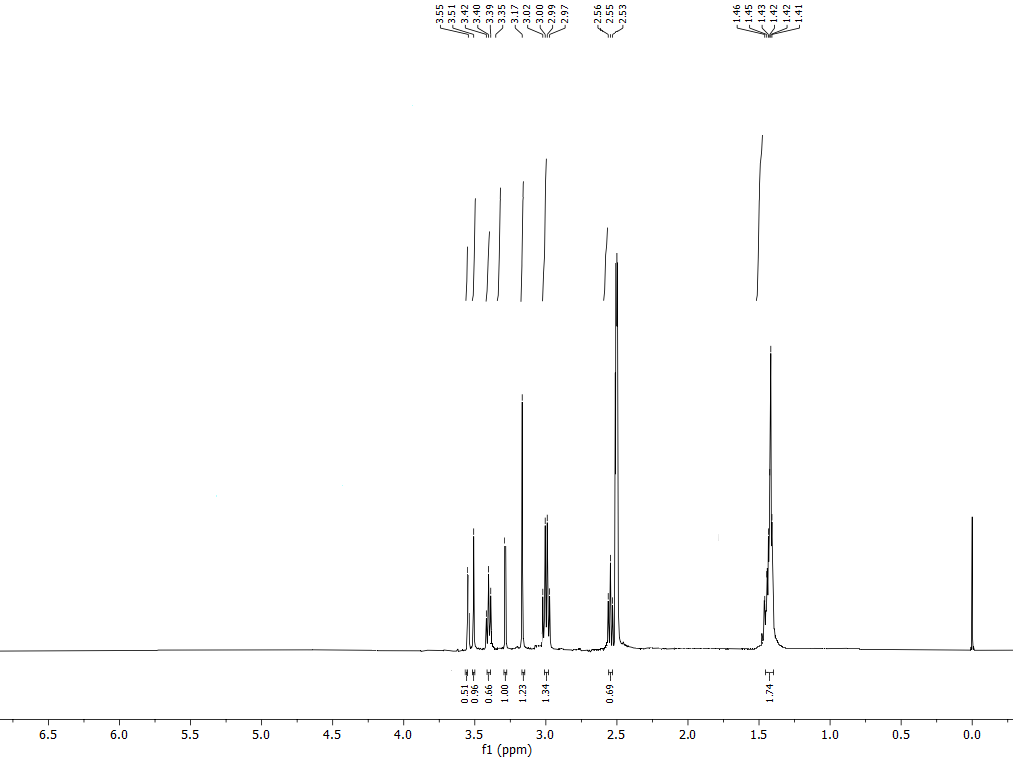
**Figure S4a.** ^1^H NMR spectrum of 8-(4-(pyrrolodine-2,5-dion)butylamino)caffeine **13**.


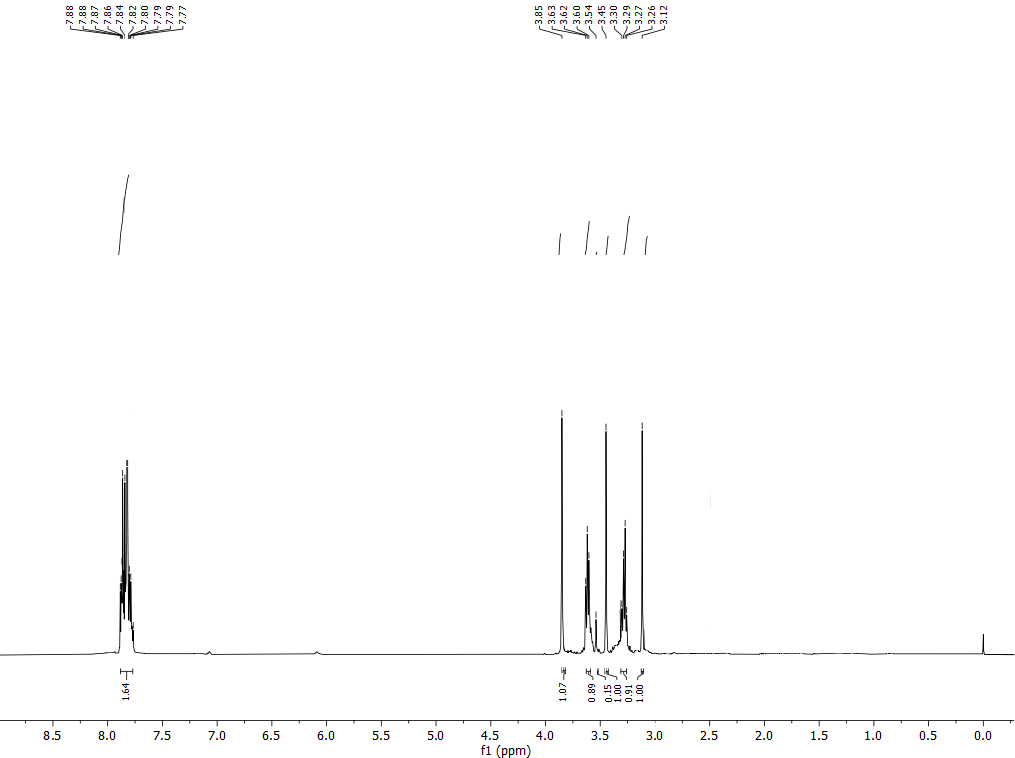
**Figure S5a.** ^1^H NMR spectrum of 8-((2-phthalimidethyl)amino)caffeine **14**.


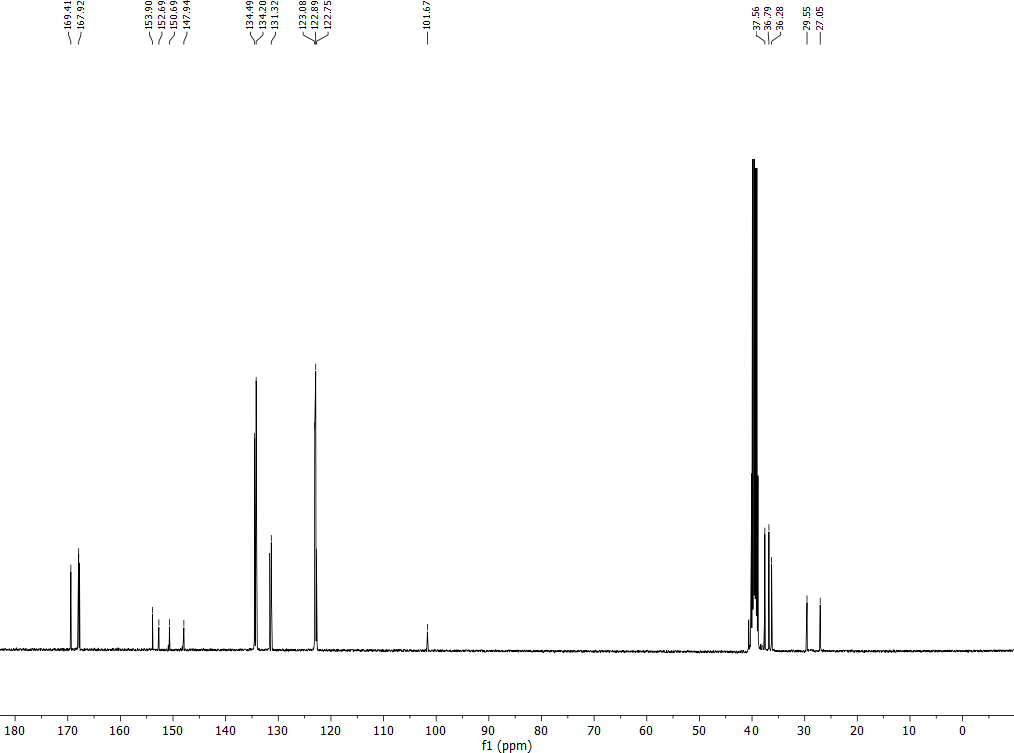
**Figure S5b.** ^1^C NMR spectrum of 8-((2-phthalimidethyl)amino)caffeine **14**.


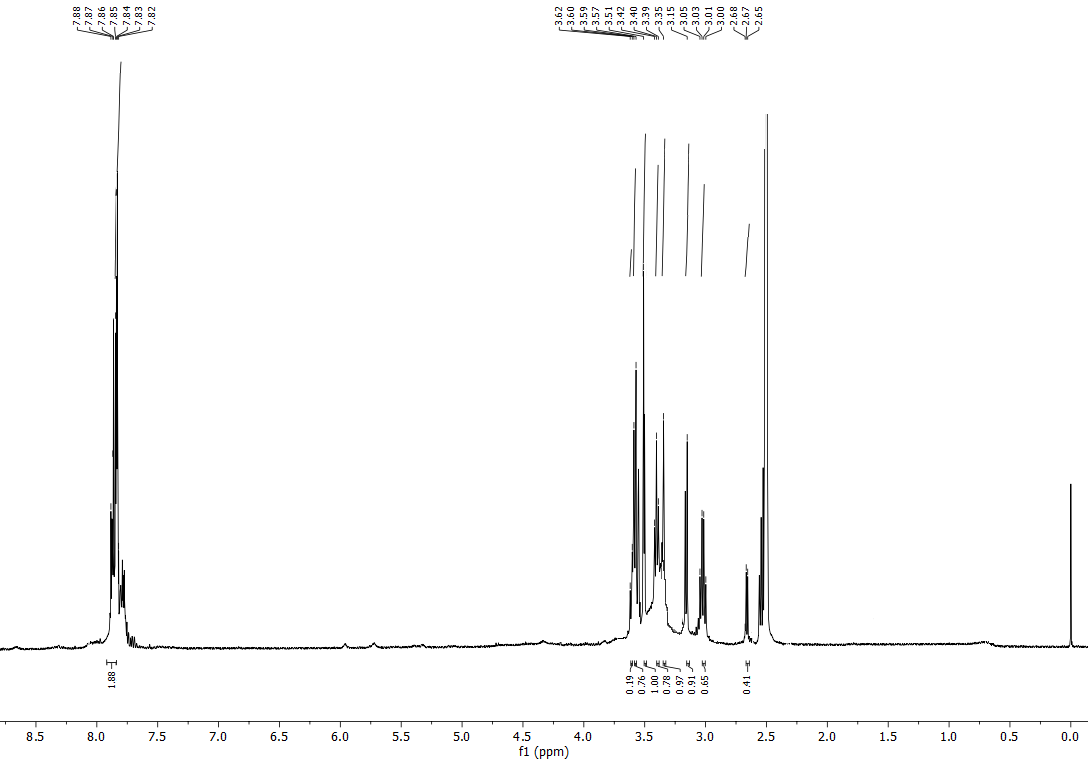
**Figure S6a.** ^1^H NMR spectrum of 8-((4-phthalimidbutyl)amino)caffeine **16**.


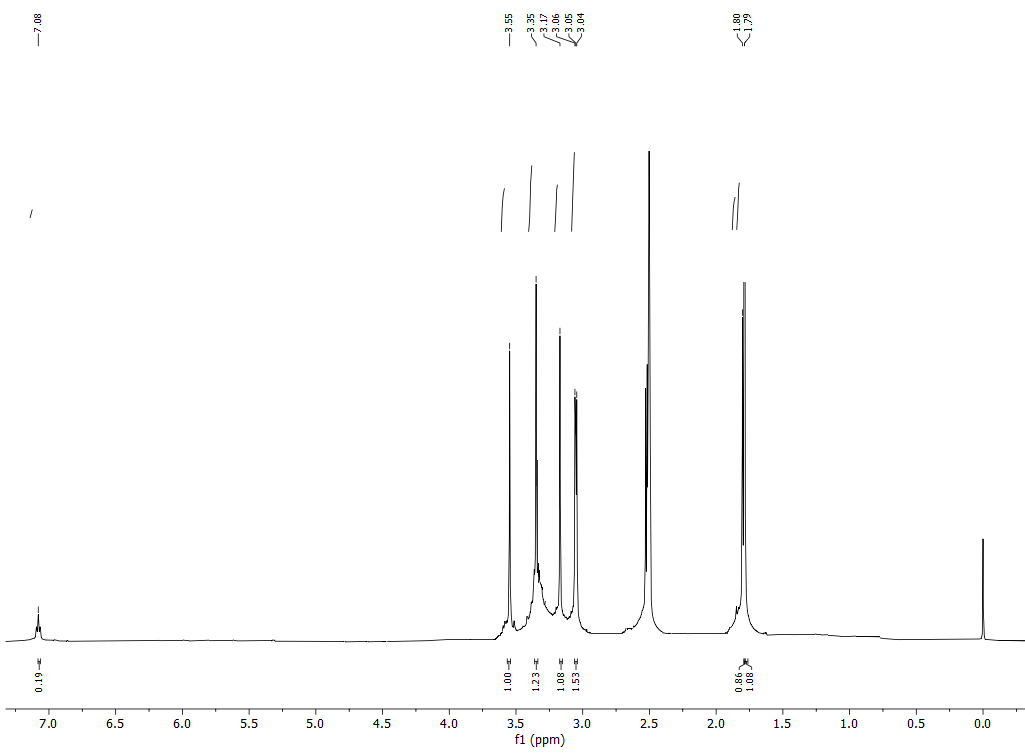
**Figure S7a.** ^1^H NMR spectrum of 8-(2-aminoethyl)-N-acetylaminocaffeine **17**.


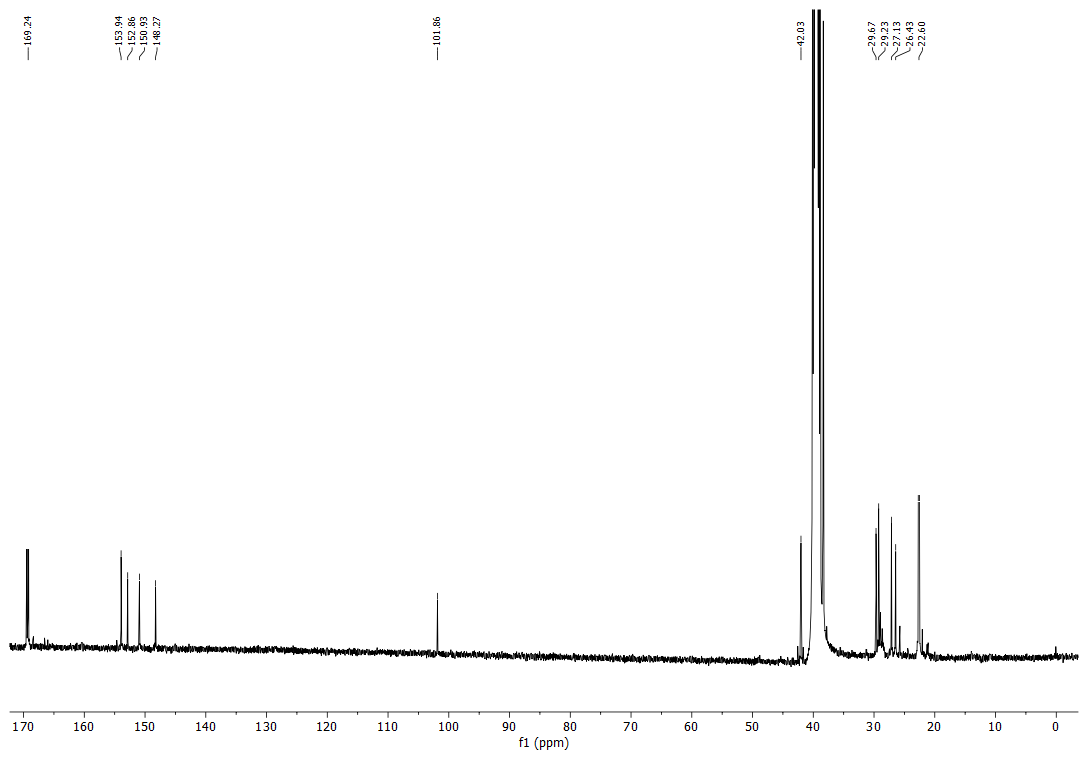
**Figure S7b.** ^13^C NMR spectrum of 8-(2-aminoethyl)-N-acetylaminocaffeine **17**.


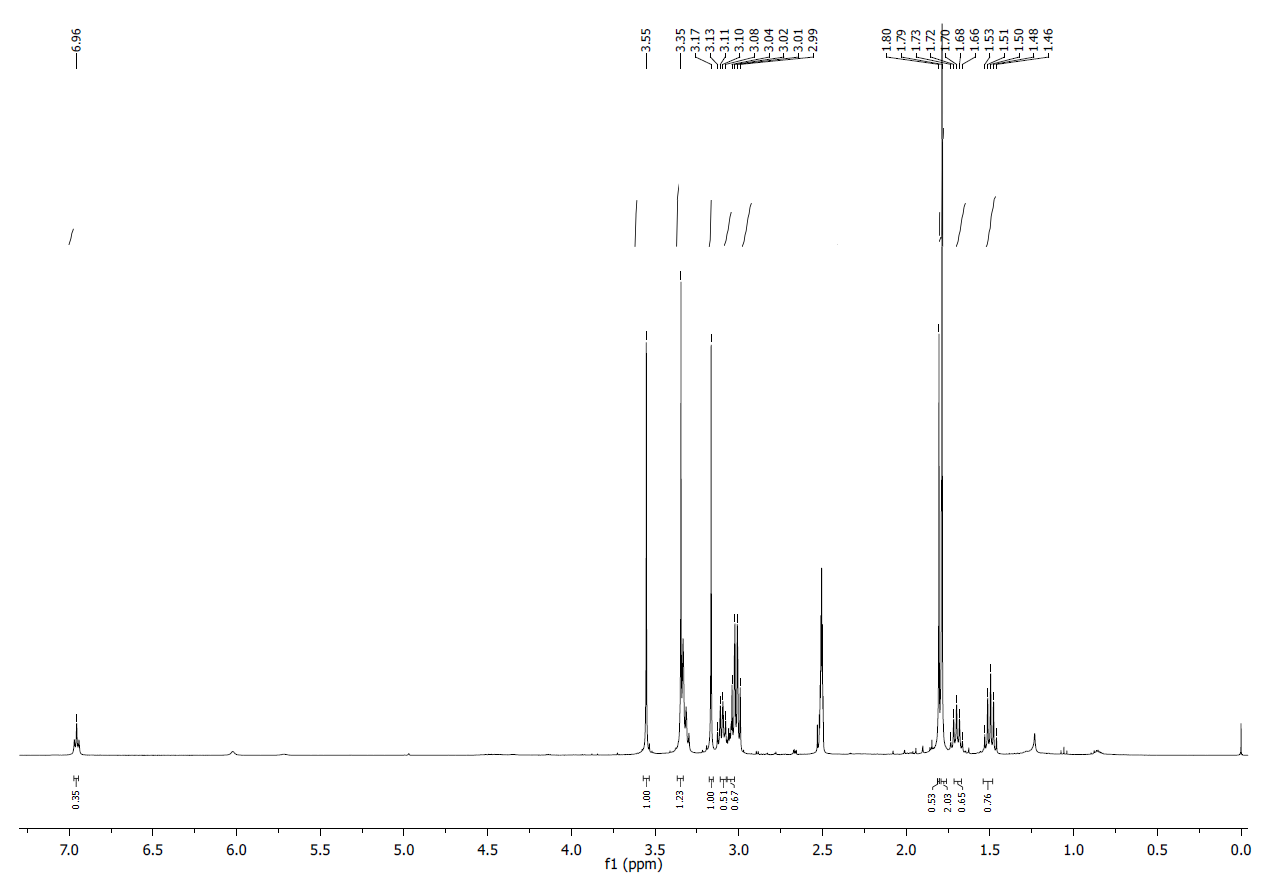
**Figure S8a.** ^1^H NMR spectrum of 8-(3-aminopropyl)-N-acetylaminocaffeine **18**.


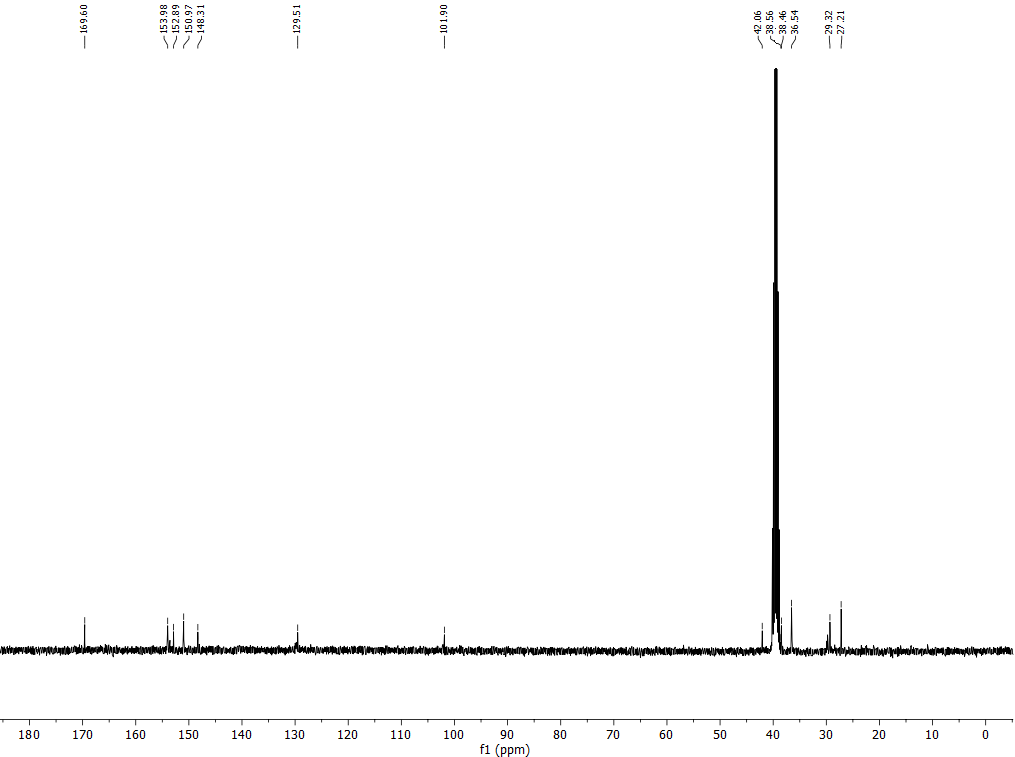

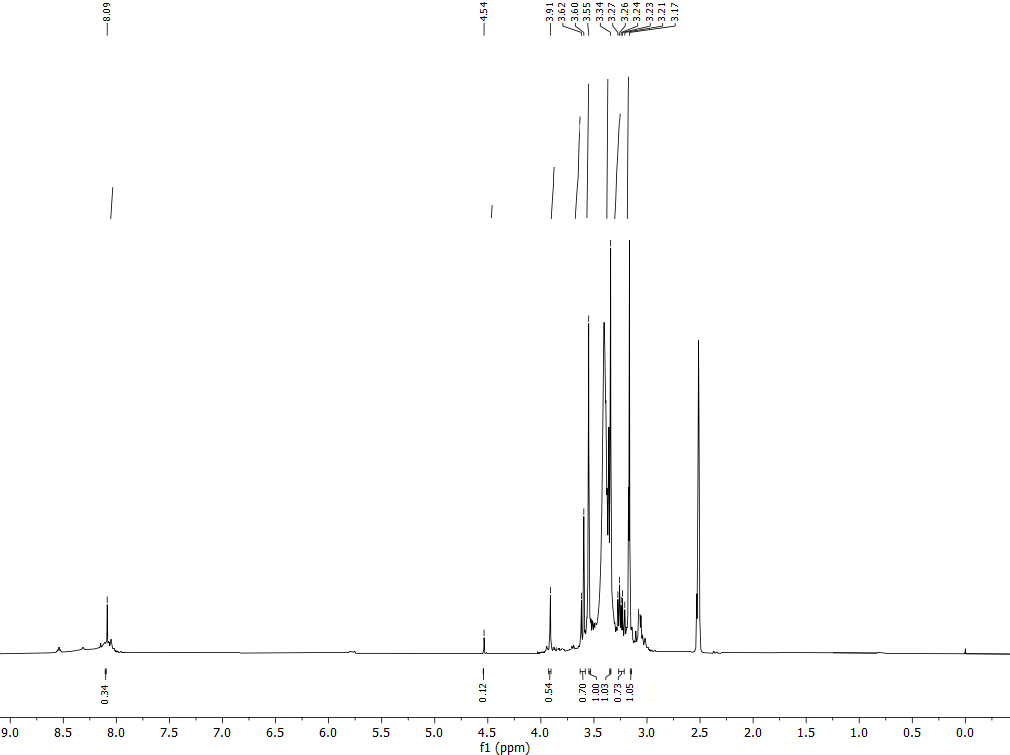

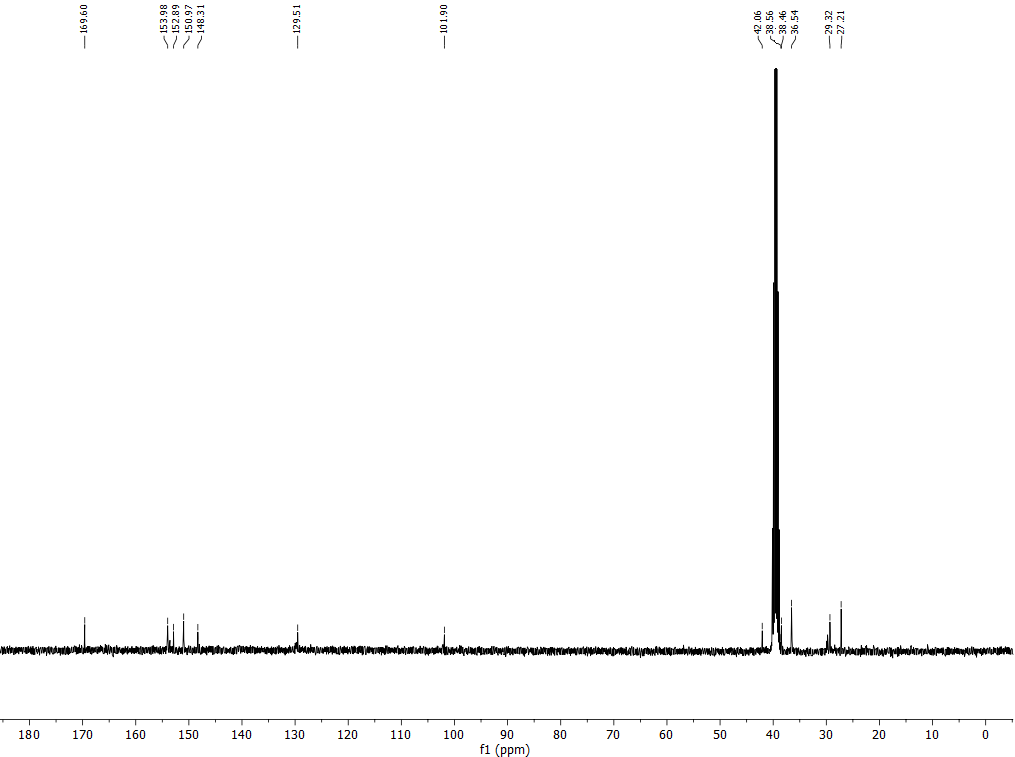
**Figure S9a.** ^1^H NMR spectrum of 8-{N-[(tiazylidyn-4-on)-2^’^-imino]ethyloamino}caffeine **20**.

**Figure S9b.** ^13^C NMR spectrum of 8-{N-[(tiazylidyn-4-on)-2^’^-imino]ethyloamino}caffeine **20**.


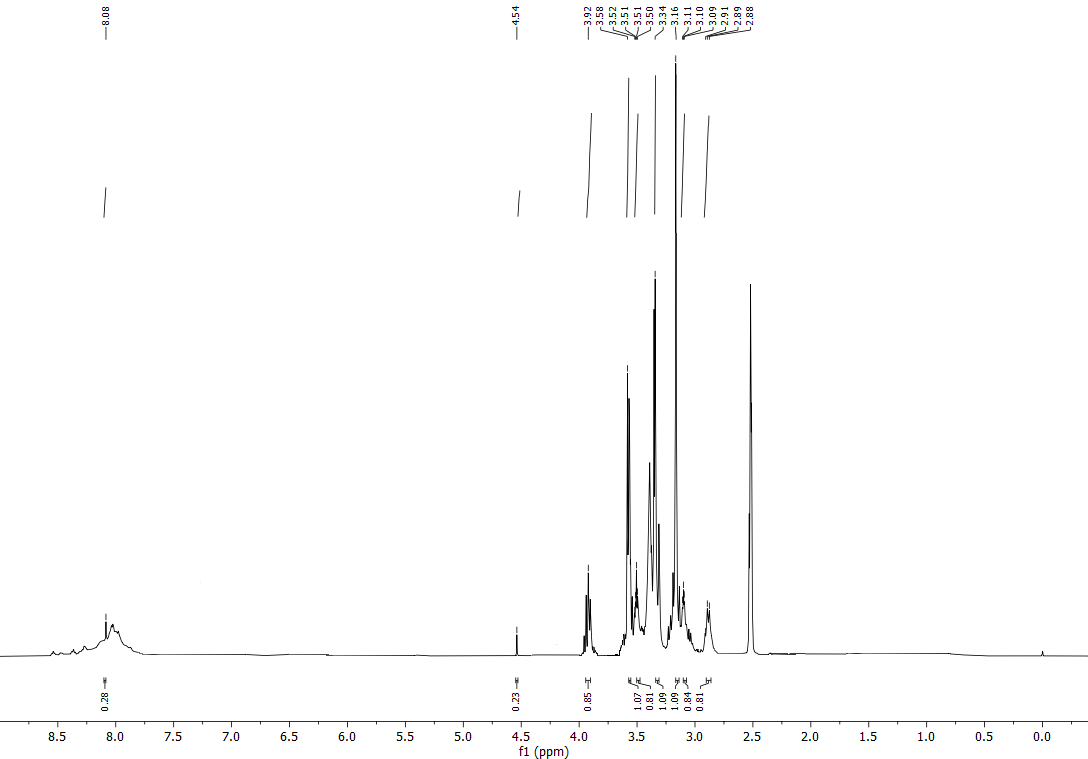
**Figure S10a.** ^1^H NMR spectrum of 8-{N-[(tiazylidyn-4-on)-3^’^-imino]propyloamino}caffeine **21**.


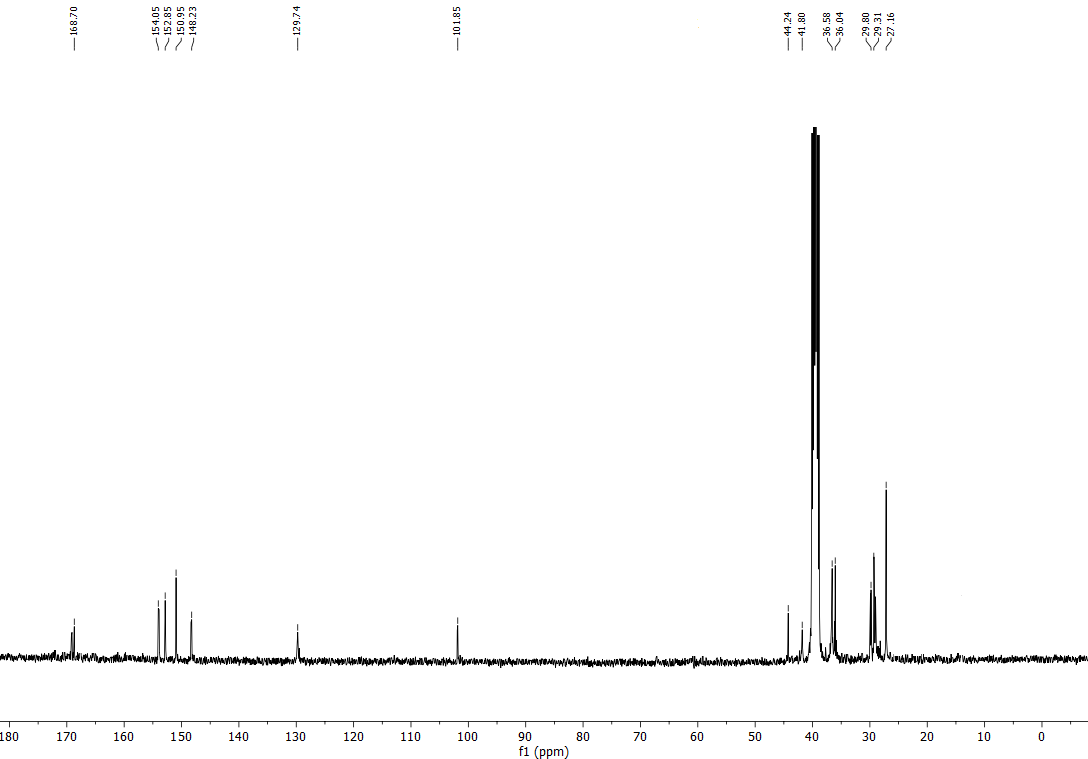
**Figure S10b.** ^13^C NMR spectrum of 8-{N-[(tiazylidyn-4-on)-3^’^-imino]propyloamino}caffeine **21**.


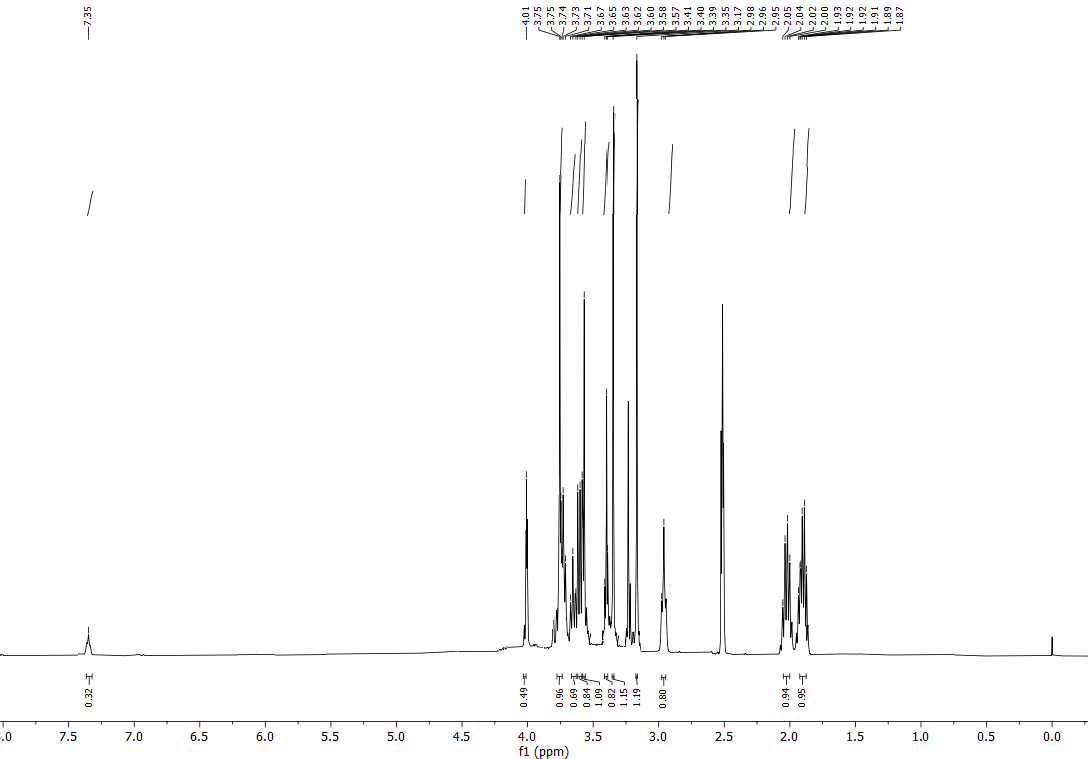
**Figure S11a.** ^1^H NMR spectrum of 8-{N-[acetylo-(pirolid-1-ylokarbotionylosulfanylo)]-aminobutyloamino}caffeine **23**.


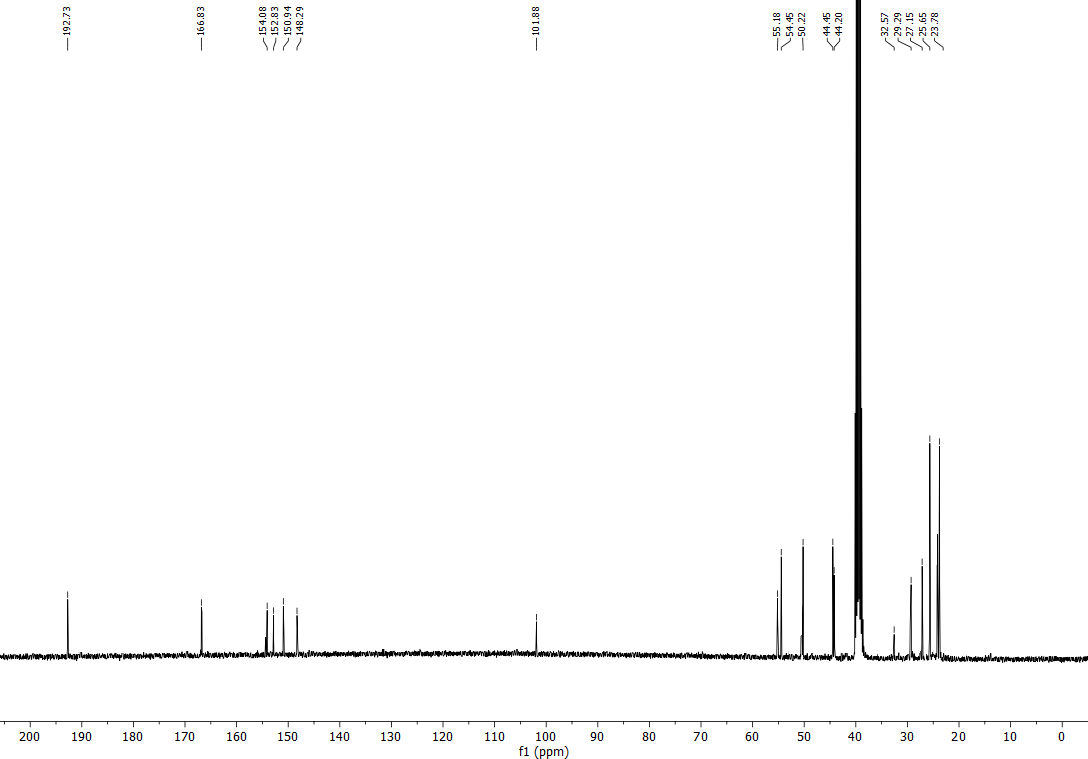


**Figure S11b.** ^13^C NMR spectrum of 8-{N-[acetylo-(pirolid-1-ylokarbotionylosulfanylo)]-

aminobutyloamino}caffeine **23**.


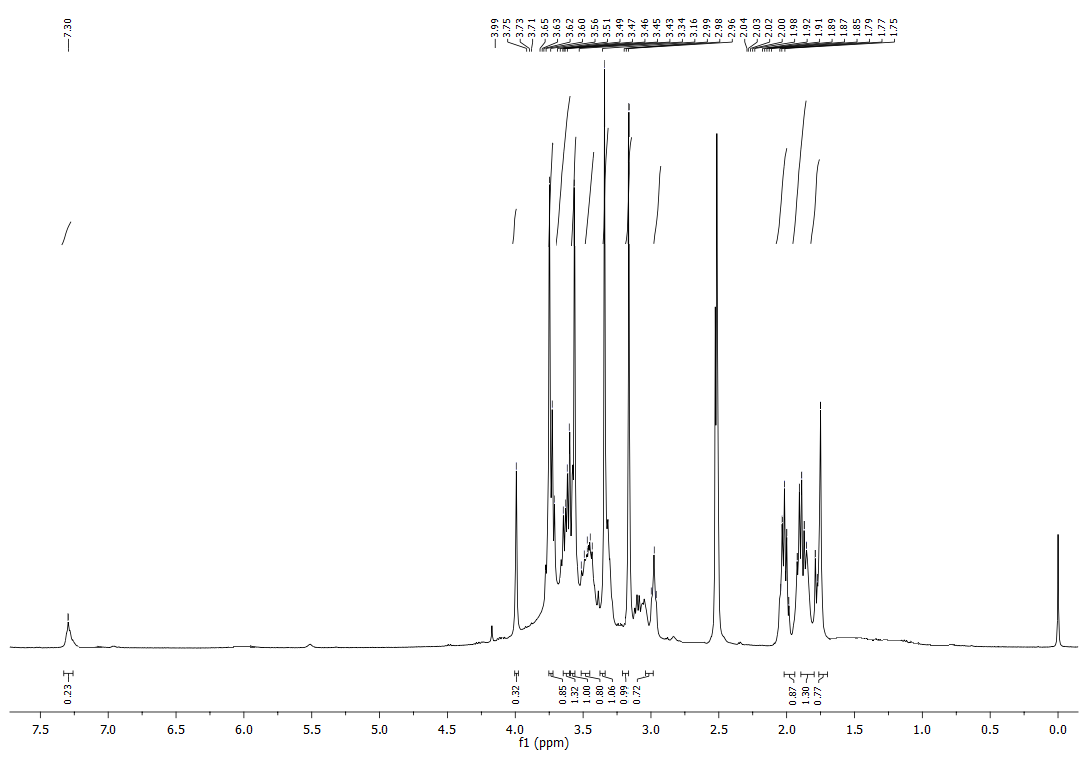
**Figure S12a.** ^1^H NMR spectrum of 8-{N-[acetylo-(pirolid-1-ylokarbotionylosulfanylo)]-aminobutyloamino}caffeine **25**.


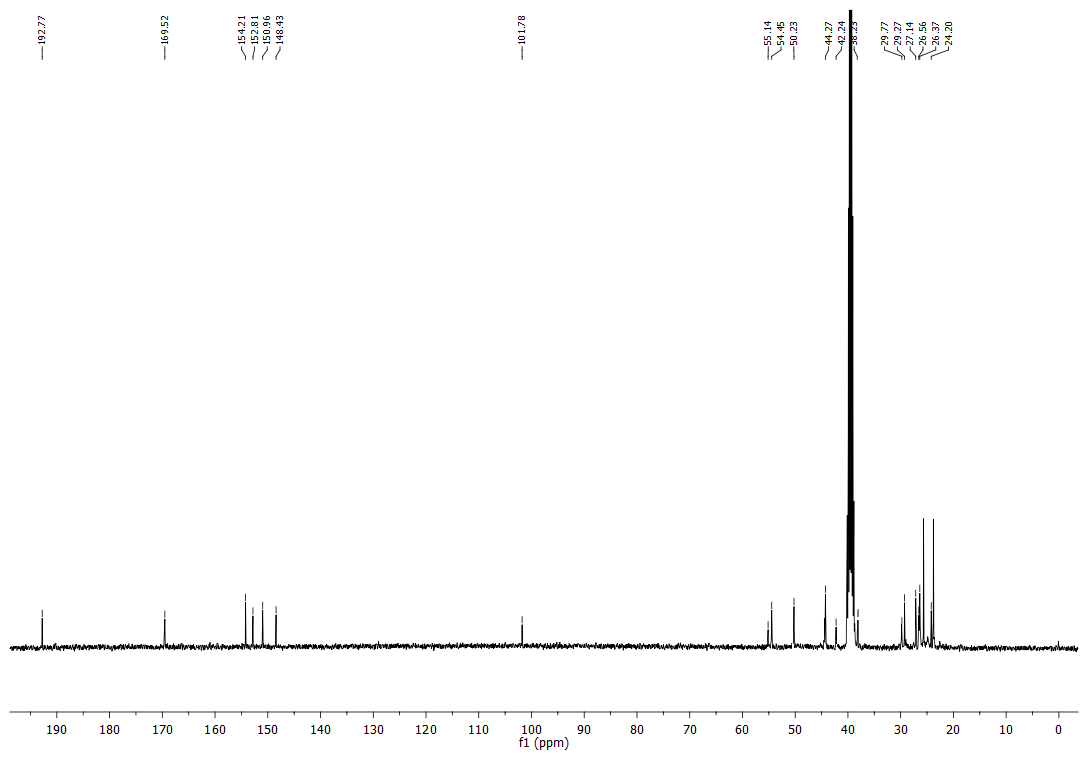


**Figure S12b.** ^13^C NMR spectrum of 8-{N-[acetylo-(pirolid-1-ylokarbotionylosulfanylo)]-

aminobutyloamino}caffeine **25**.

**
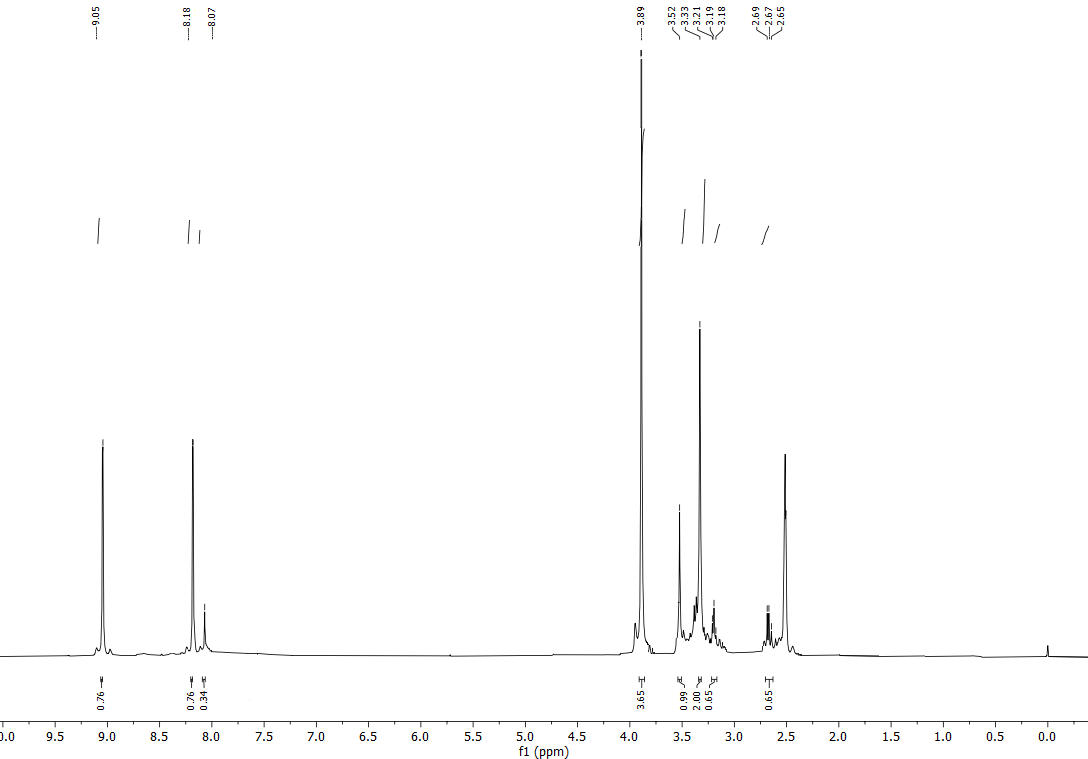
Figure S13a.** ^1^H NMR spectrum of 8-[N,N’-(1,4-dimethyl)-1,4-diaza-5,7-dienyl-8-mercapto-8-metoxycarbonyl-6-nitro-octano]caffeine **26**.


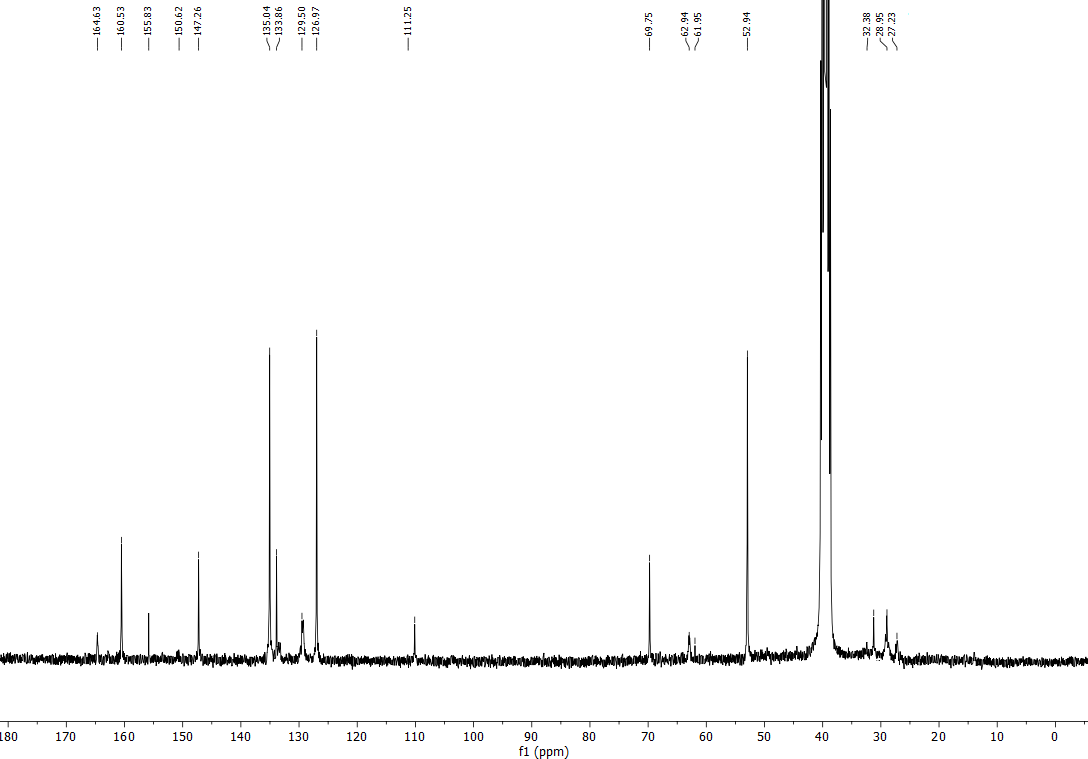
**Figure S13b.** ^13^C NMR spectrum of 8-[N,N’-(1,4-dimethyl)-1,4-diaza-5,7-dienyl-8-mercapto-8-

metoxycarbonyl-6-nitro-octano]caffeine **26**.


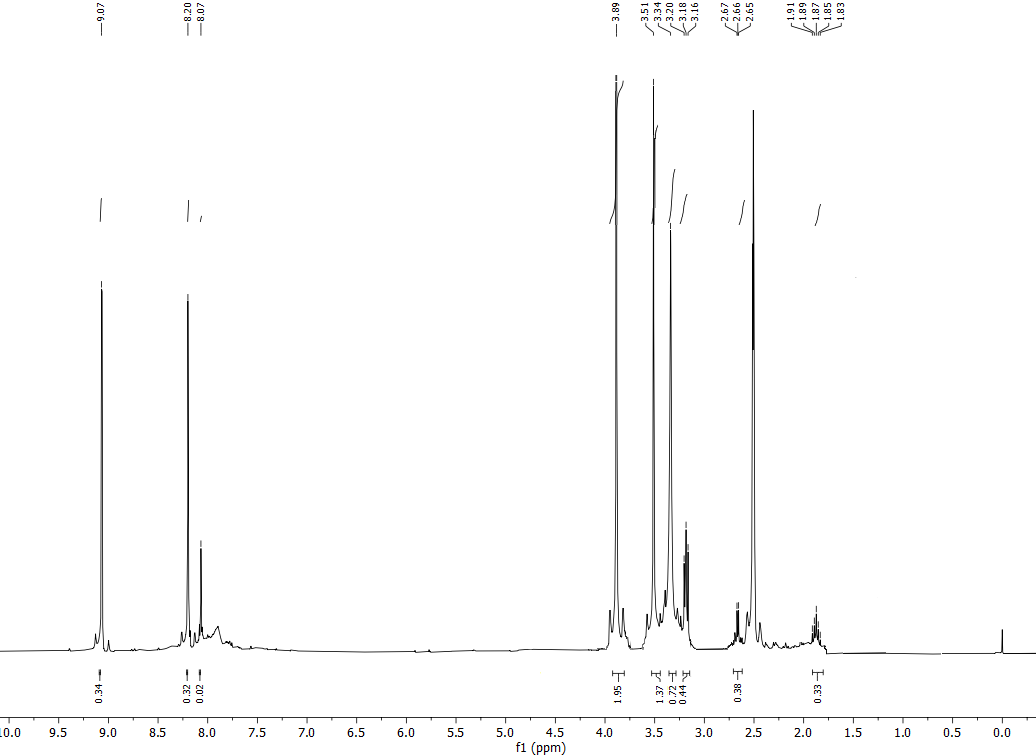
**Figure S14a.** ^1^H NMR spectrum of 8-[N,N’-(1,5-dimethyl)-1,5-diaza-6,8-dienyl-9-mercapto-9-metoxycarbonyl-7-nitro-nonano]caffeine **27**.


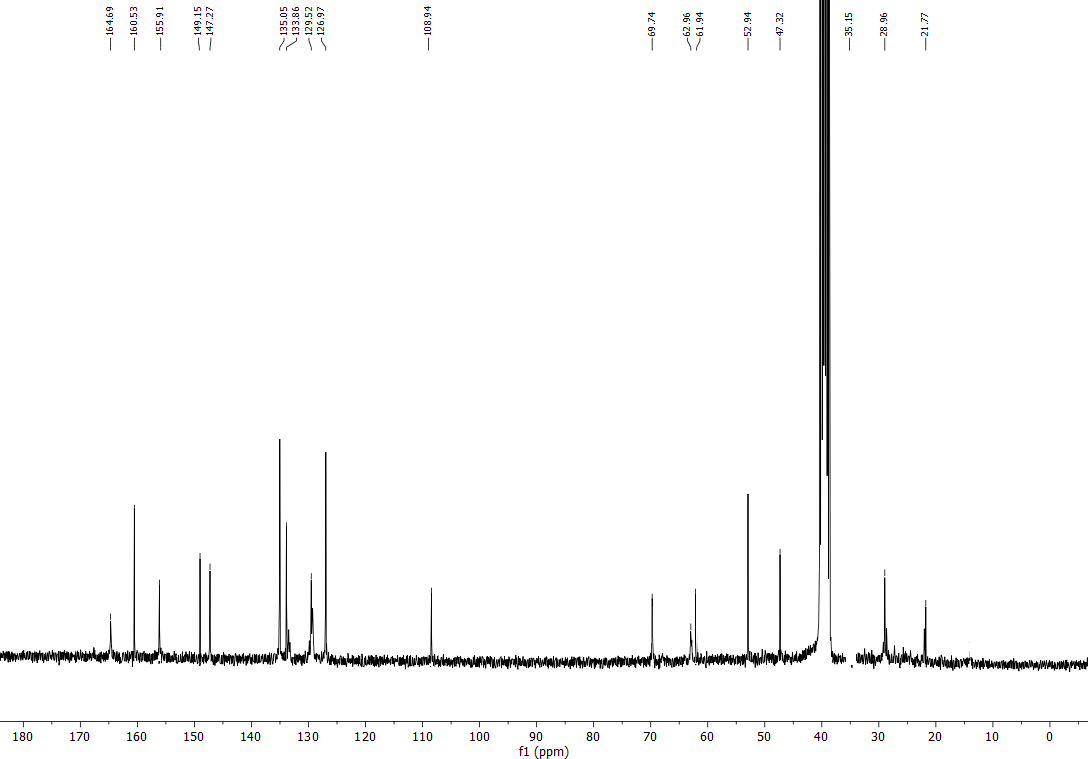
**Figure S14b.** ^13^C NMR spectrum of 8-[N,N’-(1,5-dimethyl)-1,5-diaza-6,8-dienyl-9-mercapto-9-

metoxycarbonyl-7-nitro-nonano]caffeine **27**.


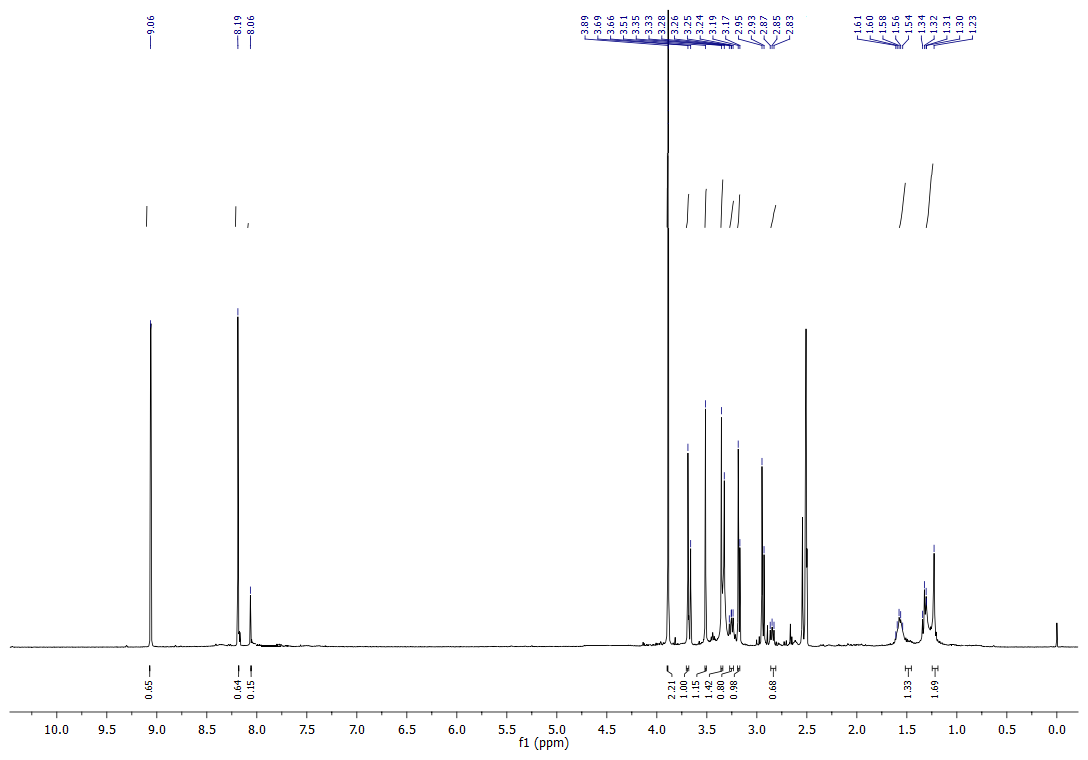
**Figure S15a.** ^1^H NMR spectrum of 8-[N,N’-(1,8-dimethyl)-1,8-diaza-9,11-dienyl-12-mercapto-12-metoxycarbonyl-10-nitro-dodecano]caffeine **28**.


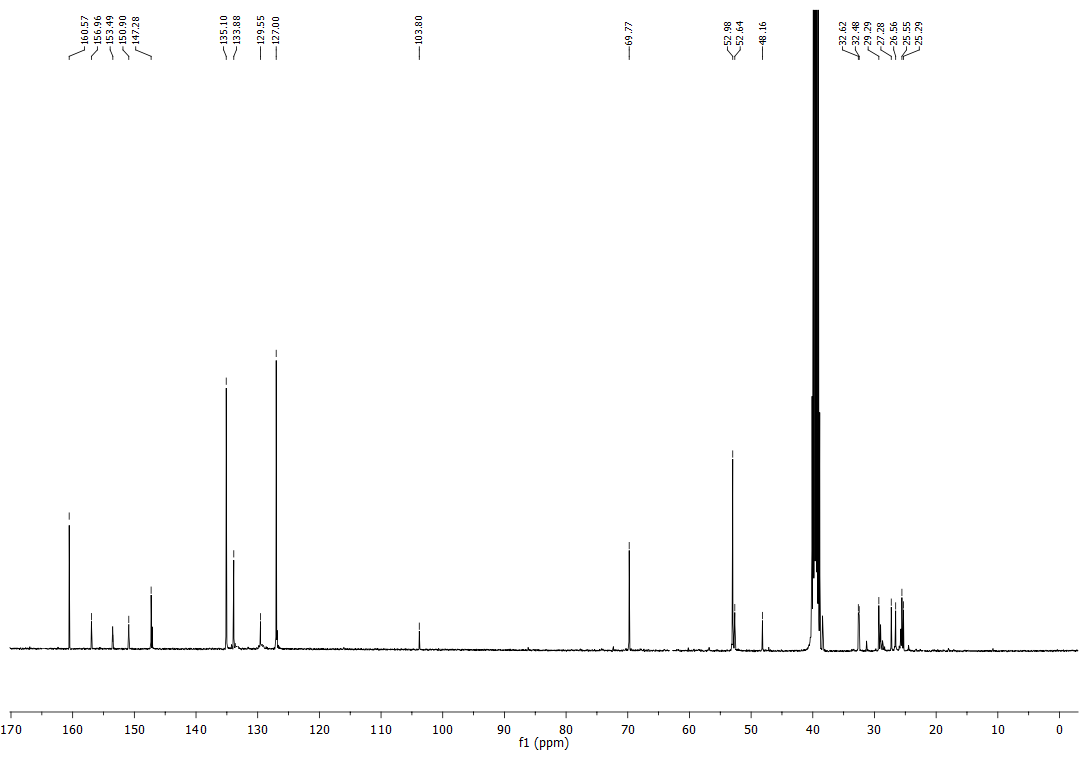


**Figure S15b.** ^13^C NMR spectrum of 8-[N,N’-(1,8-dimethyl)-1,8-diaza-9,11-dienyl-12-mercapto-12-

metoxycarbonyl-10-nitro-dodecano]caffeine **28**.


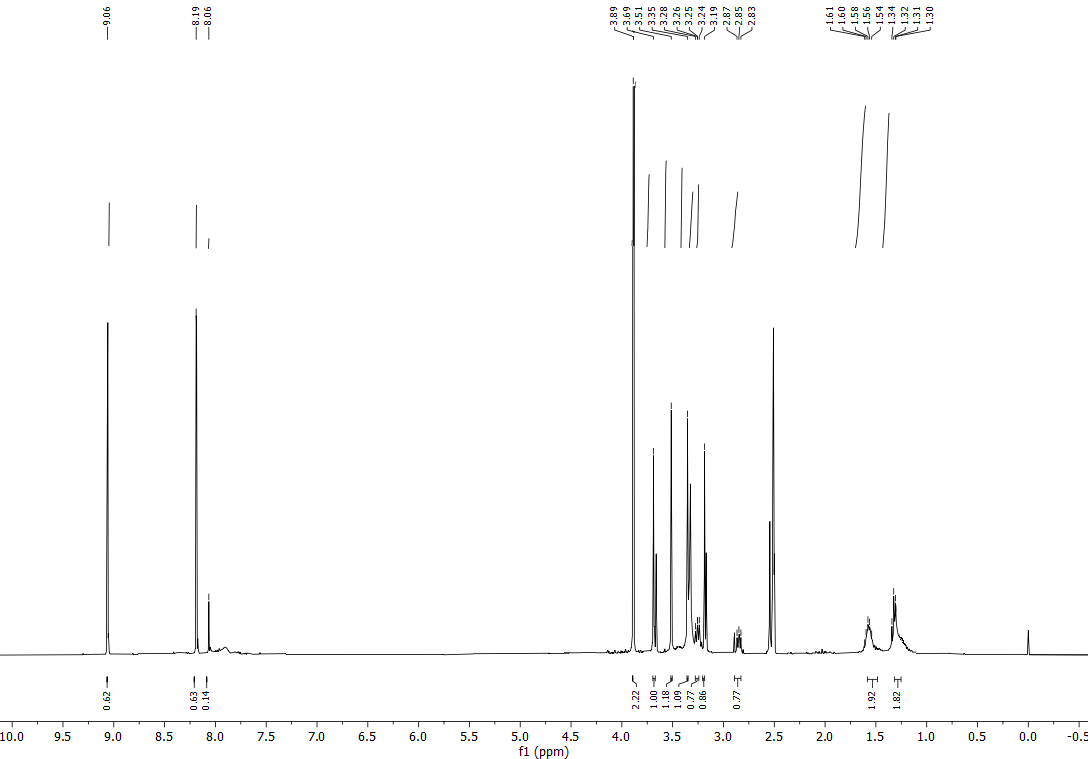
**Figure S16a.** ^1^H NMR spectrum of 8-[N,N’-(1,10-dimethyl)-1,10-diaza-11,13-dienyl-14-mercapto-14-metoxycarbonyl-12-nitro-tetradecano]caffeine **29**.


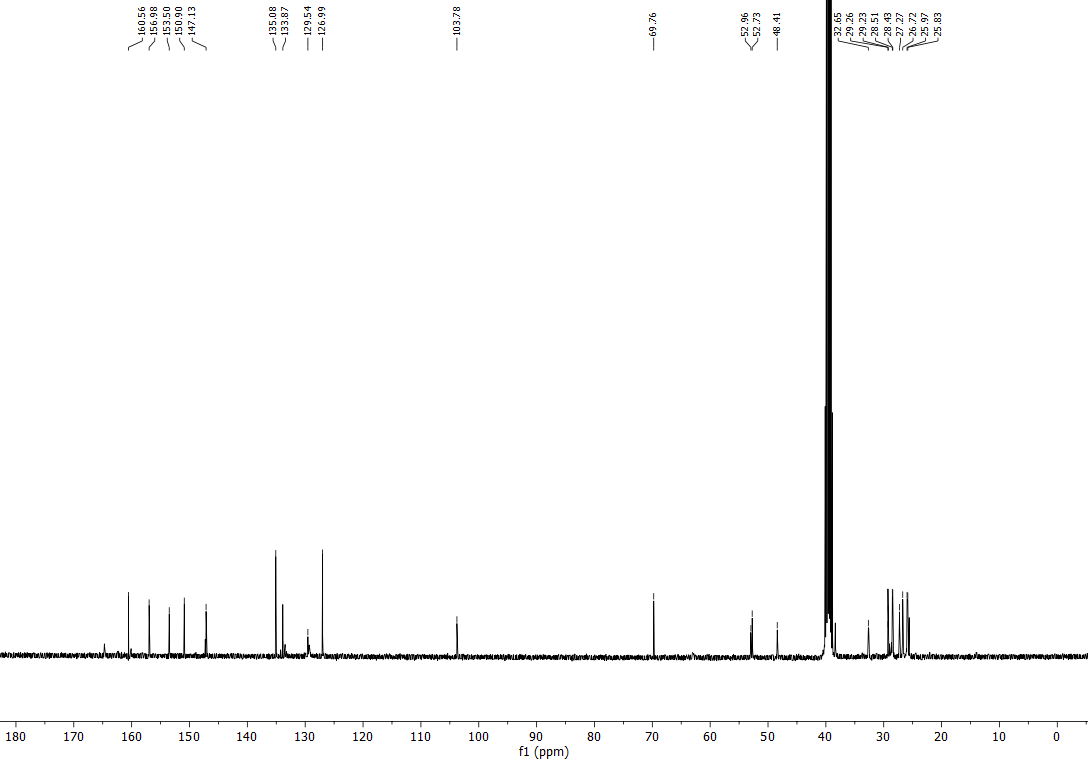
**Figure S16b.** ^13^C NMR spectrum of 8-[N,N’-(1,10-dimethyl)-1,10-diaza-11,13-dienyl-14-

mercapto-14-metoxycarbonyl-12-nitro-tetradecano]caffeine **29**.

**
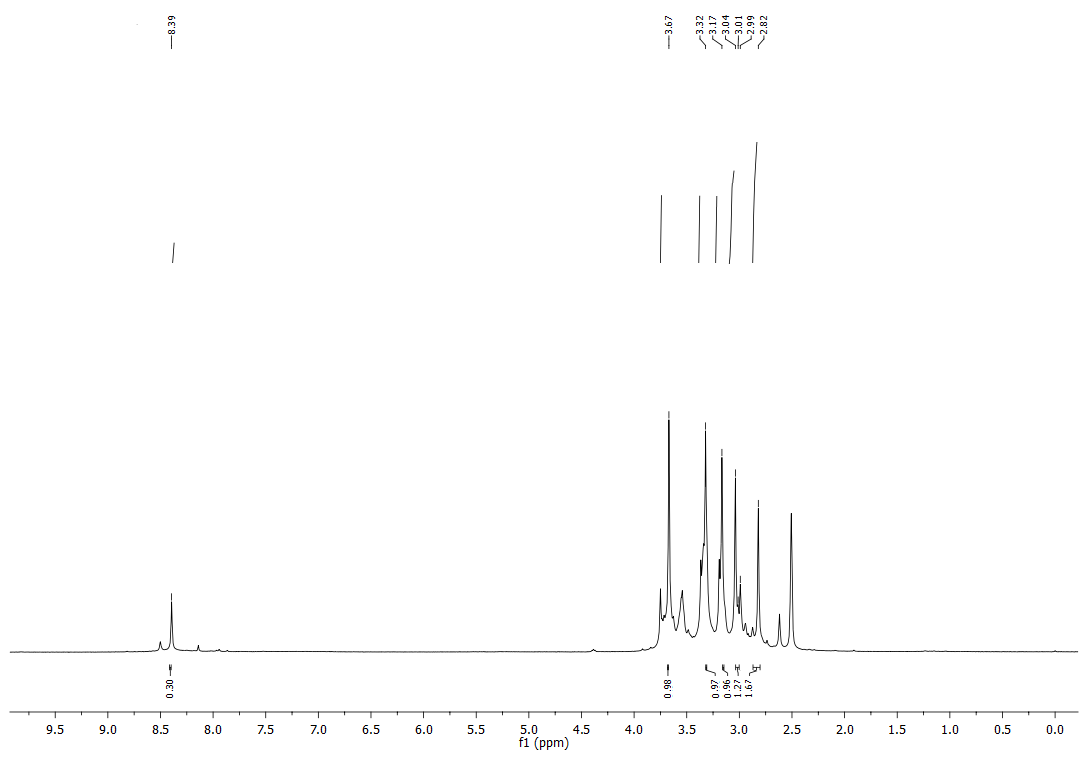
Figure S17a.** ^1^H NMR spectrum of 8,8’-[N,N,N’,N’-(1,4,9,12-tetramethyl)-5,7-dienyl-6,7-dinitro-1,4,9,12-tetraazadodecano]-dicaffeine **30**.


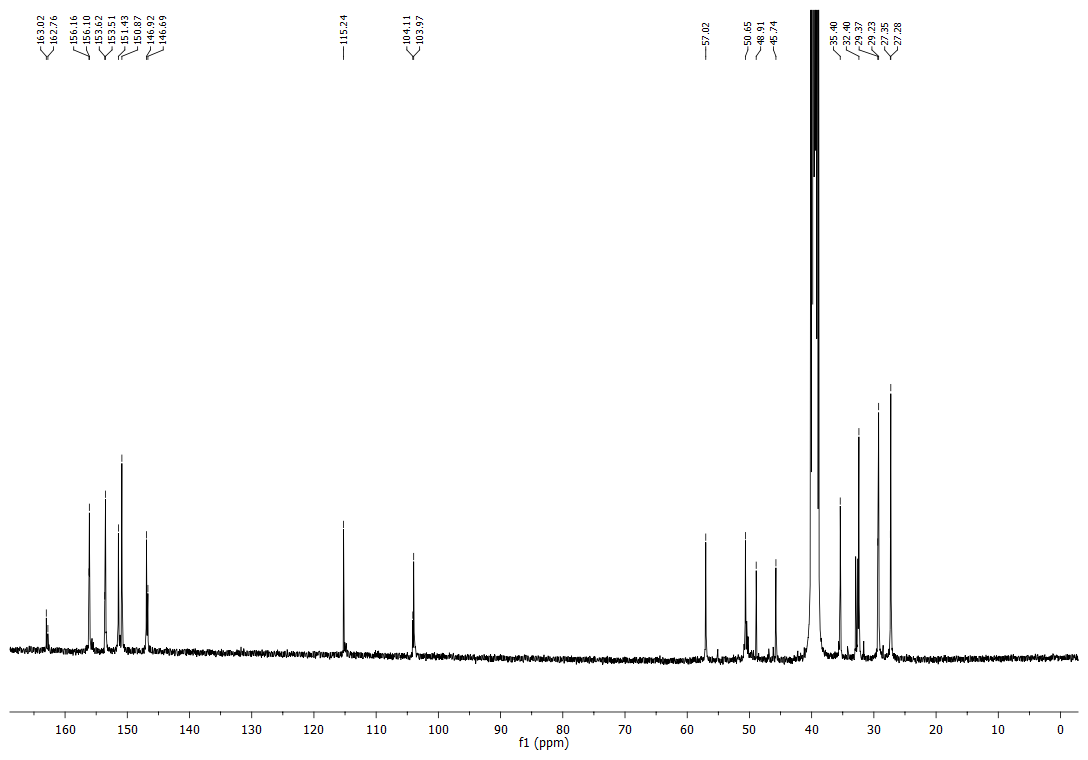


**Figure S17b.** ^13^C NMR spectrum of 8,8’-[N,N,N’,N’-(1,4,9,12-tetramethyl)-5,7-dienyl-6,7-

dinitro-1,4,9,12-tetraazadodecano]-dicaffeine **30**.
